# Sense of agency and its disturbances: A systematic review targeting the intentional binding effect in neuropsychiatric disorders

**DOI:** 10.1111/pcn.13601

**Published:** 2023-11-28

**Authors:** Lorenzo Moccia, Michelangelo di Luzio, Eliana Conte, Marco Modica, Marianna Ambrosecchia, Martina Ardizzi, Pierluigi Lanzotti, Georgios D. Kotzalidis, Delfina Janiri, Marco Di Nicola, Luigi Janiri, Vittorio Gallese, Gabriele Sani

**Affiliations:** ^1^ Department of Neuroscience, Section of Psychiatry Università Cattolica del Sacro Cuore Rome Italy; ^2^ Department of Psychiatry Fondazione Policlinico Universitario Agostino Gemelli IRCCS Rome Italy; ^3^ Child and Adolescent Neuropsychiatry Unit, Bambino Gesù Children's Hospital IRCCS Rome Italy; ^4^ Department of Medicine and Surgery, Unit of Neuroscience University of Parma Parma Italy; ^5^ NESMOS Department University of Rome La Sapienza, Faculty of Medicine and Psychology, Sant'Andrea University Hospital Rome Italy; ^6^ Italian Academy for Advanced Studies in America at Columbia University New York New York USA

**Keywords:** intentional binding, mood disorders, Parkinson's disease, schizophrenia, sense of agency

## Abstract

Sense of agency (SoA) indicates a person's ability to perceive her/his own motor acts as actually being her/his and, through them, to exert control over the course of external events. Disruptions in SoA may profoundly affect the individual's functioning, as observed in several neuropsychiatric disorders. This is the first article to systematically review studies that investigated intentional binding (IB), a quantitative proxy for SoA measurement, in neurological and psychiatric patients. Eligible were studies of IB involving patients with neurological and/or psychiatric disorders. We included 15 studies involving 692 individuals. Risk of bias was low throughout studies. Abnormally increased action‐outcome binding was found in schizophrenia and in patients with Parkinson's disease taking dopaminergic medications or reporting impulsive‐compulsive behaviors. A decreased IB effect was observed in Tourette's disorder and functional movement disorders, whereas increased action‐outcome binding was found in patients with the cortico‐basal syndrome. The extent of IB deviation from healthy control values correlated with the severity of symptoms in several disorders. Inconsistent effects were found for autism spectrum disorders, anorexia nervosa, and borderline personality disorder. Findings pave the way for treatments specifically targeting SoA in neuropsychiatric disorders where IB is altered.

The term ‘sense of agency’ (SoA) involves an individual's capacity to feel her/his own actions as actually being her/his and, through them, to exert control over the course of external events.^1^ Experiencing oneself as being the source of her/his own motor actions and not of those of others, is central to the phenomenal experience constituting self‐consciousness.^2^, ^3^ Accordingly, disruptions in SoA may profoundly affect the individual's functioning, as observed in several psychiatric and neurological disorders.

A key‐point in SoA research is how the individual's agentic experience can be operationally defined and empirically measured. Recent lines of evidence point at the multi‐faceted structure of the SoA by suggesting the existence of implicit and explicit levels of feeling and judgment of agency, respectively, that are opposed to each other; the former consists of pre‐reflective, sensorimotor processes, which are lower‐order, while the latter consist of reflective processes, which are higher‐order.^4^ The recognition of a lower‐level, pre‐reflective SoA matches the minimal self‐awareness concept, which refers to one's implicit feeling of being an immediate, invariable through time and place, subject of experience.^2^ Implicit SoA measures seek to capture this feeling by preventing individuals from explicitly thinking about agency or action control, and thus avoiding potential cognitive confounders affecting explicit SoA judgments, including response and social desirability biases.^4^ Intriguingly, a potential implicit index of SoA relies upon distortion of time perception.^5^ Haggard et al.^6^ first coined the term ‘intentional binding” (IB) to indicate an implicit, quantitative proxy for SoA measurement. IB refers to the subjective temporal contraction between a voluntary action and its outward sensory outcome. Specifically, experiments investigating the IB effect required participants to judge the time of onset of either a voluntary action or of a subsequent sensory event (e.g. an auditory tone). Voluntary actions are perceived as being shifted later in time, towards their subsequent sensory outcomes relative to control conditions, in which participants’ actions do not produce tones, and auditory tones reach perception like if they were shifted earlier, towards the actions that caused them, in comparison to control conditions involving tones but not actions. Importantly, this effect seems to occur only for voluntary actions and to be absent for passively‐induced movements, suggesting a mechanism which is specific to action intentionality.^6^

Research adopting IB paradigms has significantly enhanced our understanding of specific neurocognitive processes that may support implicit SoA. In this regard, it is possible to distinguish between two major theoretical perspectives on SoA origins, which alternatively rely upon ‘prospective’ or ‘retrospective’ mechanisms.^7^ On the one hand, SoA may arise from specific motor control mechanisms which predict an action's sensory outcomes [i.e. optimal motor control theory.^8^, ^9^ Optimal motor control requires both forward dynamic predictions, which inform the subject about the dynamics of bodily movements, and forward sensory predictions, which imply a causal relationship between actions and their sensory outcomes; this leads to predictions of the expected sensory outcomes of action based on efference copies of motor commands (i.e. neural representations of motor output signals that predict [and often modulate] sensory feedback).^8^ According to this comparator model, SoA arises from a match between predicted and actual sensory consequences of actions.^10^ On the other hand, SoA may also involve retrospective inference mechanisms that are sustained by the actual presence of the sensory outcomes of action (i.e. apparent mental causation theory^11^). As a consequence, action intentionality occurs if a thought/intention about action precedes action, if it is consistent with action, and is the only possible cause of the action.^11^ While the optimal motor control model emphasizes the importance of ‘internal’ predictive signals in generating SoA, the apparent mental causation theory highlights the role of inferential processes that rely on ‘external’ contextual signals. However, as suggested by Moore et al.,^12^ the dichotomy between forward prediction and retrospective inference may not capture the full complexity of SoA. Indeed, SoA and IB may depend upon a weighted integration of multiple agency cues, including sensorimotor predictions and external outcomes of actions, together with prior beliefs. The relative significance of these cues is determined by their reliability, with the more reliable source of information providing a stronger influence on the agentic experience.^13^

Multiple neural structures have been linked to distinct aspects of the agency experience or specific stages of the process leading to SoA. This collection of neural structures involved in agency‐related processes is extensive and encompasses various regions, including the dorsolateral prefrontal cortex (DLPFC), the cingulate cortex (CC), the supplementary and pre‐supplementary motor areas (SMA and pre‐SMA), motor cortex and premotor cortex, the posterior parietal cortex (PPC) and inferior parietal lobule (IPL), the insula, as well as the precuneus.^14^ The presence of multiple neural structures involved in agency‐related processes and their distribution across the entire brain likely reflects the complexity of the phenomenon and the various methodological and experimental approaches used to investigate its different facets.^15^ The involvement of these structures is a result of the contribution of multiple mechanisms that facilitate the coupling of behavior with mental states and sensory effects.^16^ Indeed, the various mechanisms underlying agency‐related processes, along with their corresponding neural structures, can be attributed to overarching functions, including monitoring sensorimotor congruence and multimodal integration, the elaboration and implementation of intentions, action monitoring, and the attribution of ownership and agency.^14^

According to other lines of evidence, the ongoing resting state pattern of activity in self‐referential brain regions may affect SoA at a brain network background.^17^ In healthy individuals, when mental contents are externally‐oriented, an increased activity in the lateral regions of the Central Executive Network (CEN) has been observed. Conversely, when mental contents are internalized, there is heightened activity in the Default Mode Network (DMN).^18^ Importantly, the predominance of either external or internal mental contents in awareness appears to take place with a reciprocal balance (i.e. when there is increased activity in DMN and the focus is on internal mental contents, there is a corresponding decrease in activity in the CEN, and vice versa).^19^, ^20^

Several studies based on non‐invasive stimulation techniques attempted to precisely localize brain areas underlying IB. Moore et al.^21^, ^22^ investigated the effect of theta‐burst stimulation over the pre‐SMA. The inhibition of the activity of the pre‐SMA via theta‐burst stimulation was found to lead to a significant reduction in IB. In addition, there is evidence for pre‐SMA involvement in determining the subjective time compression between voluntary actions and subsequent sensory outcomes in studies adopting the IB paradigm.^23^ Of note, a recent meta‐analysis that investigated the effect of anodal stimulation over the PFC, highlighted the role of the DLPFC in increasing the IB effect in free‐selection action tasks, thus suggesting a precise contribution of DLPFC to SoA in action‐selection processes.^24^

The acquisition of an intact SoA is regarded as a milestone in cognitive development and human evolutionary adaptation.^25^, ^26^ Experiencing abnormal SoA can hinder behavioral performance on cognitive tasks,^27^ lead to decreased awareness,^28^ and adversely affect everyday quality of life and mental health,^29^ as in the case of several neuropsychiatric conditions.^7^ Categorical approaches to psychopathology usually consider individual disorders as distinct entities, each with its own signs, symptoms, and etiology. In contrast, ‘transdiagnostic’ avenues in neuropsychiatric research take into account the opportunity offered by considering distinct disorders as variable alterations of the cognitive systems that underpin them, including SoA.^30^, ^31^, ^32^ Given the putative link between IB and SoA, IB offers a useful paradigm to address this opportunity. In this regard, studies on IB have highlighted many substantial inter‐individual differences in SoA, as well as their implications for mental health. For instance, while healthy individuals exhibit a stronger prospective contribution to SoA as measured through IB,^33^ increased schizotypal traits have been linked with reduced prospective and increased retrospective IB.^34^ Similarly, IB seems to vary between individuals with high and low hypnotic susceptibility^35^ and correlates with narcissistic personality traits.^36^ Furthermore, the magnitude of prospective IB is associated in healthy individuals with the degree of inter‐modular connections of a fronto‐parietal module that includes the pre‐SMA, the IPL, and the dorsal precuneus.^37^ Taken together, this evidence points at inter‐individual differences in the propensity to experience an enhanced or reduced IB, both at a behavioral level and in terms of brain activity and is consistent with a quantitative approach to nosography whereby different psychopathological phenotypes may exist on a continuum of SoA under‐ or over‐ascription.

With this background in mind, this article aims to review systematically those studies that investigated IB in patients with neurological disease and/or psychiatric disorders.

## Methods

### Search strategy

We conducted a systematic research on the PubMed/MEDLINE database and on the ClinicalTrials.gov site from inception to 18‐November‐2022, using ‘IB’ as search strategy (details of search strategy are provided in supporting material). We adhered to the 2020 Preferred Reporting Items for Systematic reviews and Meta‐Analyses (PRISMA) statement.^38^ Our above‐reported search produced 200 results. After applying exclusion criteria, 15 articles remained **(**Table 1 **)**. Results are shown in the PRISMA flowchart (Fig. S1 in supporting material). Detailed information about each study regarding inclusion or reason for exclusion can be found in Table S1 in supporting material. Risk of bias (RoB) of included studies was assessed using the Cochrane RoB 2.0 tool^39^ (Table S2 in supporting material).

**Table 1:** Summary of included studies

Study	Population	Design	Results	Observations
Haggard et al.^40^	8 pts. with SCZ (6 ♂; 2 ♀; age x̄ = 44.6 ± 9.9) vs 8 HCs (6 ♂; 2 ♀; age x̄ = 42.25 ± 9.3)	LC paradigm with composite binding measure calculation	SCZ pts. show ↑ IB as showed a ↑ composite (action + tone) shift. HCs showed a moderate IB with only action binding and no tone binding	Pts with schizophrenia show an exaggerated version of the normal binding effect, or hyperbinding. This pattern suggests an implicit over‐attribution of sensory consequences of movement to oneself in this population which may ultimately lead to abnormal SoA and typical delusional symptoms
Franck et al.,^41^	24 pts. with SCZ (−5 lost for exaggerated SD) (14 ♂; 5 ♀; age x̄ = 33 ± 10) vs 24 HCs (age x̄ = 29 ± 7)	LC paradigm modified with a somatic sensory event instead of a tone. IB calculated on passive movement time estimation shift. Four blocks: (i) baseline, (ii) sequence (iii) agent, (iv) other	SCZ group show ↑ IB effect in sequence, agent and other conditions with ↑ anticipatory shift of passive movement time judgment. This effect was particularly important for the sequence (2) condition. HCs showed anticipatory effect only in sequence (2) condition	Results show that hyperbinding effect is found for somatic sensory events such as passive movements, as well as auditory events. SoA could be altered not only by increasing the attribution of agency to effects voluntarily produced but also to passively induced effects
Moore et al.,^22^	9 pts. with PD (7 ♂; 2 ♀; age x̄ = 65.11 ± 8) vs 9 HCs (3 ♂; 6 ♀; age x̄ = 62.00 ± 6)	LC paradigm with composite binding measure calculation. PD pts. were tested both on and off medication	Overall binding in PD pts. off‐medication is not significantly different to HCs. This suggests that PD itself is not associated with abnormal action awareness or SoA. However overall binding in PD pts. on medication is significantly ↑ than in PD pts. off‐medication	Dopaminergic medication enhanced the experience of agency in PD pts. Consequently, the dopamine network could have a role in SoA production and its dysregulation to aberrant agency attribution
Voss et al.,^42^	24 pts. with SCZ (22 ♂; 2 ♀; age x̄ = 34.8 ± 13.1) vs 24 HCs (21 ♂; 3 ♀; age x̄ = 34.4 ± 10.9)	LC paradigm modified with probability conditions (75% and 50%). Only the action time estimation is calculated in the different conditions	Binding effects is significantly ↑ in pts. than in HCs. HCs show a ↑ predictive component of IB, and only minimal retrospective contribution. In the SCZ group, action binding towards tones do not vary with the actual predictability of the tone but was equal in 50 and 75% tone frequency conditions (↑ retrospective component). In particular positive symptoms are associated with a ↓ predictive component.	These findings are consistent with neurocognitive theories that emphasize the role of impaired sensorimotor predictions in the genesis of positive symptoms in SCZ. SCZ pts. appear to be driven mostly by sensory external cues with a ↓ capacity to predict, especially when positive psychotic symptoms are present
Hauser et al.,^43^	30 pts. with PP (19 ♂;11 ♀; age x̄ = 26 ± 6) vs 30 HCs (18 ♂;12 ♀; age x̄ = 29 ± 6)	LC paradigm modified with probability conditions (75% and 50%). Only the action time estimation is calculated in the different conditions	↑ IB in PP compared to HCs. Binding effects were numerically greater in PP pts. than in HCs, although this difference was not statistically significant. PP pts. show significantly ↑ predictive and retrospective influences on SoA compared with HCs. Follow‐up t‐tests suggest that this was most pronounced for the predictive component	Results consistent with PP pts. intermediate between HCs and SCZ pts. Hyper‐prediction in PP and hypo‐prediction in SCZ consistent with studies highlighting neurochemical changes associated with disease progression
Kranick et al.,^44^	20 pts. with CD (8 ♂; 12 ♀; age x̄ = 46.3 ± 12.4) *vs* 20 HCs (8 ♂; 12 ♀; age x̄ = 46.5 ± 11.7)	LC paradigm modified with an initial conditioning where auditory tones are paired with emotional faces. Composite measure calculation	No main effect of group, interaction or valence on action‐ binding. Main effect of group for tone‐binding, pts. ↓ binding than HCs. CD pts. show ↓ overall IB compared to HCs. No effect of affective valence on binding in either HCs or pts. with motor CD	Motor symptoms with a sense of ‘loss of control’ on movements with hyper‐motility could be linked to a reduced SoA. Similarity between CD and GTS in IB results
Sperduti et al.,^45^	15 pts. with ASD (AS or HFA or PDD‐NOS) (age x̄ = 33.53 ± 11.01) vs 17 HCs (age x̄ = 33.06 ± 11.13)	IE paradigm with auditory, visual or multimodal sensory stimulus. Two conditions: control and operant. Intervals lasting 250, 450, 650 ms. A PDS has been used as a measure of IB. The more positive the PDS the greater the IB effect	The HCs group show a PDS significantly different from zero in all conditions. In ASD group PDS is not significant in ViIB condition for any interval delay, showing altered IB. In AuIB and MuIB, PDS was ↑ for the 450 and 650 ms intervals, showing an altered IB in the 250 ms interval for these two latter conditions. Main result is that adults with ASDs showed ↓ IB, as compared to HCs	The multimodal condition did not elicit a true multimodal integration process, and the auditory signals may have driven subjects' performance
Wolpe et al.,^46^	10 pts. with CBS (6 ♂; 4 ♀; age x̄ = 69 ± 10) vs 16 HCs (10 ♂; 6 ♀; age x̄ = 64 ± 7)	LC paradigm with composite binding measure calculation. The task is completed with both hands (less affected and most affected by symptoms). Subsequent scanning with structural MRI, diffusion MRI and functional resting state MRI	CBS markedly have ↑ IB in the most‐affected hand. The less affected hand does not differ from HCs. ↑ action binding in more affected hand is related +ve with severity of alien limb symptoms and –ve with apraxia scores. Action binding in the most‐affected hand significantly have a + ve correlation with GM volume in the pre‐SMA and correlates with WM deficit in several areas, including white matter adjacent to the pre‐SMA and PFC, the superior longitudinal fasciculus, and the anterior CC, as well as with FC between the pre‐SMA and medial and lateral PFC, including DLPFC	Association between sense of ‘extraneity’ in body perception and ↑ action binding in CBS somehow similar to SCZ. The pre‐SMA may serve as a critical hub within a frontal network for awareness and control of voluntary action. Changes in pre‐SMA and its frontal connections might affect both the objective capacity for voluntary control of action, and the subjective experience of agency. Further studies needed to highlight the role of pre‐SMA in disorders with SoA alterations
Graham‐Schmidt et al.,^47^	39 pts. with SCZ (passivity‐ve: n 24: 18 ♂; 6 ♀; age x̄ = 43.1 ± 1.8; passivity +ve: n 15: 8 ♂; 7 ♀; age x̄ = 42.8 ± 2.5) *vs* 43 HCs (23 ♂; 20 ♀; age x̄ = 44.6 ± 1.7)	IE paradigm with composite binding calculation. Three blocks: (i) active; (ii) passive, movement hided; (iii) other: movement visible. Intervals lasting 200, 400 or 600 ms.	As the interval ↑, SCZ pts., regardless of passivity symptom profile, perceive the interval to be shorter compared to HCs. In SCZ pts. passivity –ve, the perceived interval was significantly longer for both active and other conditions compared to the passive condition. There was no significant difference between active and other conditions. SCZ pts. passivity +ve did not perceive a difference in the interval between active, passive or other conditions at any of the intervals presented, thus not displaying differences in perceiving their own movement or a passive or others' movements	A difference in SoA perception may exist between SCZ pts. with different symptomatology. Passivity symptoms could suggest a loss of capacity to discriminate voluntary actions from passive movements or others' actions. SCZ without passivity could lead to inability to discriminate between voluntary and others' action, maintaining perception for passive movements
Saito et al.,^48^	19 pts. with PD (11 ♂;8 ♀; age x̄ = 66.0 ± 6.2) vs 25 HCs (12 ♂;13 ♀;age x̄ = 64.9 ± 2.9) Pts participated in the experiment while under regular dopaminergic medication	LC paradigm with separate calculation of action and tone timing. Task performed with the hands of both the more affected side and the less affected side of motor symptoms	HCs show IB effect. Relative to HCs, PD pts. displayed significantly ↓ action binding, whereas no significant differences between the two groups were observed for tone binding. No significant effect of hand was detected in both operant conditions	PD pts. on regular dopaminergic medication have only tone binding. In PD pts. action binding could be influenced by –ve feelings about their motor competences
Ricciardi et al.,^49^	19 pts. with PD‐ICB (12 ♂;7 ♀: age x̄ = 53.6 ± 9.3) vs 19 PD‐no‐ICB (11 ♂;8 ♀: age x̄ = 56.9 ± 8.4) vs 19 HCs (8♂;11 ♀: age x̄ = 52.6 ± 7.4)	LC paradigm with composite binding measure calculation	Significant difference in action‐binding between PD‐ICB and HCs and between PD‐ICB and PD‐no‐ICB, with PD‐ ICB showing a ↑ action binding effect. No differences between PD‐no‐ICB and HCs at action‐binding. No differences between groups at tone‐binding	ICBs may be a relatively common side‐effect in pts. taking dopamine agonists for PD and involve a weakened subjective experience of volition associated with actions suggesting an aberrant SoA
Möller et al.,^50^	21 pts. with BPD = 21 (4 ♂; 17 ♀; age x̄ = 28.4 ± 8.8) vs 21 HCs (4 ♂; 17 ♀; x̄ = 30.3 ± 9.9)	IE paradigm incorporated into an aRHI task. Four experimental conditions: congruent and incongruent with respect to the position of the artificial hand, agency and non‐ agency with respect to pressing the button	No one of the four experimental condition results have significance at statistical analysis	Although the time intervals were clearly underestimated in all experimental conditions, which clearly indicate the expected subjective compression of time, no significant group effect, nor any interaction effect were found. SoA Self‐reported questionnaire showed ↑ SoA
Zapparoli et al.,^51^	25 pts. with GTS (20 ♂; 5 ♀; age x̄ = 26.3 ± 9.4) vs 25 HCs (12 ♂; 13 ♀; age x̄ = 25.7 ± 3.8]	IE paradigm during fMRI. Visual sensory stimulus. Two conditions: passive and operant	IB effect only in HCs in operant conditioning at 200 ms of latency. ↓ IB in GTS pts. respect to HCs (only at 200 ms). Severity of motor tics (YGTSS scores) inversely correlated with IB for intentional acts. Activation of several brain regions at fMRI in HCs at 200 ms latency but not in GTS, specifically, left pre‐SMA, left preGy, superior pLob, postGy, IC, bilateral cerebellum, left Hip and bilateral superior fGy	GTS is a ‘loss of control’ and hyper‐motility disorder showing pattern similarity to CD
Finnemann et al.,^52^	23 pts. with ASD (11♂; 12♀; age x̄ = 29.0 ± 6.1) vs 25 HCs (10 ♂; 15 ♀; age x̄ = 31.2 ± 5.7)	LC paradigm, modified, with probability conditions (75% and 50%). Only action time estimation is calculated in the different conditions	No significant differences between the two groups with both participants with ASD and HCs displaying ↑ IB in 75% effect probability conditions compared to 50% effect probability conditions	In ASD, like in health conditions, the predictive component of IB with respect to the retrospective one appears to be conserved
Engel et al.,^53^	22 ♀ active AN pts., age x̄ = 29.9 ± 10.6 (range 19–63 years); vs 30 ♀ recovered AN pts., age x̄ = 32.5 ± 10.6 (range 19–56 years); vs 29 ♀ HCs, age x̄ = 30.3 ± 14.0 (range 18–55 years)	LC paradigm, modified, participants reporting on white screen background to action or tone (an answer screen appeared, asking to report at what time did they press the key, or they heard the tone; the answer box logged numbers 0–60 only). Participants were asked to judge time in 4 randomly presented conditions (2 BL [tone presentation by experimenters or keypress by participant at will], 2 operant [auditory tone presented 250 ms after pressing the key]) with 28 (instead of the classical 40) trials in each, 3 practice, 25 analyzed	Mixed ANOVA showed a significant main effect of Event and a significant interaction effect of Condition × Event in the whole group, without differences between the groups. AN pts. had ↓ compared to HCs; pts. with active AN had ↑ state anxiety than recovered women with AN. State anxiety scores (as assessed through the STI‐Y1) did not predict IB. Groups did not differ on their explicit sense of control as assessed through the SCS. After correcting for false discovery rate, implicit SoA (assessed through SCS) correlated inversely with tone shift only in recovered AN pts., and explicit feeling of control correlated inversely with state anxiety in recovered AN pts. and HCs, but not in active AN pts	Implicit sense of control SoA in active and recovered AN pts. is not altered using an IB task. Contrary to expectations, state anxiety did not predict IB, and consequently, SoA. Patients with active disorder have more state anxiety and experience a loss of control (↓SoA)

Abbreviations: AN, anorexia nervosa; aRHI, active rubber hand illusion; ASD, Autism Spectrum Disorder; AS, Asperger Syndrome; AuIB, auditory intentional binding; BL, baseline; BPD, Borderline personality disorder; CBS, Cortical‐Basal Syndrome; CC, corpus callosum; CD, conversion disorder; DLPFC, dorso‐lateral prefrontal cortex; fMRI, functional magnetic resonance imaging; FC, functional connectivity; fGy, frontal gyrus; GM, gray matter; GTS, Gilles de la Tourette Syndrome; HAS, High Functioning Autism; HCs, healthy controls; Hip, hippocampus; IB, intentional binding; IC, insular cortex; ICB, impulsive‐compulsive behavior; IE, interval estimation; LC, Libet's clock; MRI, magnetic resonance imaging; ms, milliseconds; MuIB, Multimodal Intentional binding; PD, Parkinson's Disease; PDD‐NOS, Pervasive Developmental Disorder not otherwise specified; PDS, proportion difference score; PD‐ICB, Parkinson's disease with impulsive‐compulsive behavior; PD‐no‐ICB, Parkinson's disease without impulsive‐compulsive behavior; pts., patients; PFC, prefrontal cortex; pLob, parietal lobule; postGy, postcentral gyrus; PP, putative prodrome of psychosis; preGy, precentral gyrus; SCS, Self‐Control Scale; SCZ, schizophrenia; SMA, Supplementary Motor area; SoA, sense of agency; STAI‐Y1, State–Trait Anxiety Inventory, Y1 version‐State; ViIB, visual intentional binding; WM, white matter; x̄, mean; YGTSS, Yale Global Tic Severity Scale; ±, standard deviation (SD); ♀, female; ♂, male; ↓, decreased, drop; ↑, increased. −ve, negative; +ve, positive.

## Results

### Overview of the included studies

Eligible studies dealt with data from 357 patients with neuropsychiatric disorders matched with 335 HCs. Of included patients, 95 were with schizophrenia (SCZ), 30 with a putative prodromal psychosis (PP), 21 with borderline personality disorder (BPD), 66 with Parkinson's disease (PD), 38 with an autism spectrum disorder (ASD), 29 with functional movement disorders (FMDs), 25 with Gilles de la Tourette syndrome (GTS), 52 with anorexia nervosa (AN; 22 with active disorder and 30 after they had recovered), and 10 with Cortico‐Basal syndrome (CBS).

**Fig. 1:**
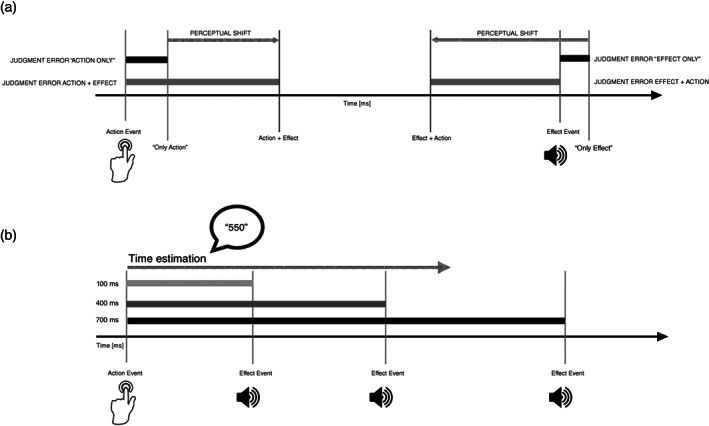
Temporal binding and timing judgments according to Haggard's et al. (2002) procedure (i.e. Libet Clock Task) and to Interval Estimation (IE) Task. (a) Libet Clock Task: In baseline conditions participants report the position of the clock hand at which they either perform a key presses (i.e. ‘action only’), or, in a separate block, at which they heard the onset of a sensory event, usually an auditory tone (i.e. ‘effect only’). In operant conditions, 250 ms following the key press, the effect, identical to the stimulus that is presented in the baseline condition, occurs. Perceptual shifts are calculated by subtracting mean judgment errors (i.e. the difference between the actual time the event occurred and the time it was perceived to occur) in baseline conditions, where participants pressed the key without producing the sound, or heard the sound without pressing the key, from mean judgment errors for the same event in the operant condition. (b) IE Task: In this task participants are instructed to perform a single key press, at a time of their choosing. After the key press, a subsequent sensory event is presented with different time delay. Following the sensory event, participants are asked to estimate the perceived action‐outcome delay between the onset of the key press and the onset of the effect.

Temporal binding, including timing judgments, was calculated in 11 studies using variations of the experimental procedure introduced by Haggard et al.^6^ In studies using this methodology, participants are expected to estimate the timing of their actions and subsequent events with the so‐called ‘Libet Clock’ (LC), which is presented on a screen. A full rotation of such clock's hand takes about 2560 ms rather than 60 s.^54^, ^55^ The shifts in action and sound judgments are usually combined in a composite binding measure quantifying the global subjective temporal association between actions and their sensory consequences (Fig. 1).

Four studies utilized a different paradigm,^45^, ^47^, ^50^, ^51^ namely interval estimation (IE), which involved the direct evaluation of the interval between an active or passive action and a subsequent sensory outcome that is presented with different time delay. IB is derived from relatively shortened IEs in operant conditions compared to baseline (Fig. 1). Results derived from the tasks vary depending on the neuropsychiatric diagnosis assessed. Therefore, we divided the following section in paragraphs according to the neuropsychiatric disorder/disease investigated.

### Schizophrenia spectrum and other psychotic disorders

SCZ is usually defined by the presence of three main clusters of symptoms: positive (e.g. hallucinations and delusions), negative (e.g. blunted affects, social withdrawal, anhedonia) and disorganized symptoms (e.g. disorganized speech and behavior),^56^ with disordered cognition crossing all the above dimensions.^57^ Many studies shed light on the neurobiological basis of these symptoms, pointing to the role of structural and functional brain abnormalities as well as neurotransmitter system dysfunctions.^58^, ^59^ Atypical perceptions of agency, which are relatively common in SCZ, typically fall within the positive symptom domain and may encompass a wide range of manifestations.^60^ Among these, the most prevalent are passivity symptoms, often referred to as delusions of control. Studies suggest that specific problems with sensorimotor predictions, which are crucial for SoA, may determine action awareness abnormalities in SCZ.^61^ Indeed, it has been hypothesized that failures in the attribution of self‐agency to the sensory consequences of actions might result from a deficit within the comparator mechanism, either related to the generation of inadequate internal predictions and/or to an impaired comparison with the actual sensory afference.^62^, ^63^, ^64^ Another line of evidence stems from research conducted on sensory attenuation (i.e. the phenomenon whereby self‐initiated sensory inputs are perceived with less intensity, compared to externally generated sensory inputs). Self‐generated stimuli are less attenuated in individuals with SCZ compared to HCs, thus suggesting an impairment in predicting the sensory consequences of an action as well as in labeling movements as self‐generated.^65^

#### IB studies in SCZ spectrum and other psychotic disorders

Five studies evaluated IB in patients with a disorder in the psychotic spectrum. Haggard et al.^40^ assessed IB in eight patients with SCZ compared to eight HCs. Timing judgments were obtained adopting a computerized version of the LC. Patients with SCZ displayed an increased IB effect relative to HCs. Specifically, a positive shift for action judgments coupled with a negative shift for the auditory tone judgment was observed in the SCZ group, thus suggesting that the temporal interval between action and sensory outcome was significantly shorter for patients, compared to controls.^40^

Franck et al.^41^ evaluated IB in 19 SCZ patients matched with 24 HCs with a modified version of Haggard's et al.^6^ method. The current experiment was designed to test whether abnormal IB would extend in SCZ to judgments of somatic events, such as passive movements. Participants were asked to place their index fingers of both hands on two button boxes, on which a magnetic solenoid was installed. Activating the magnetic solenoid through the computer resulted in the corresponding button moving downward under the participant's passive finger pressure. Participants were instructed to judge the subjective time of onset of the fall of their right index finger induced by the solenoid in four different conditions, by verbally reporting the perceived LC hand's position when the right index finger began moving passively. In baseline trials, the left button and hand remained still throughout, whereas the right response button moved downward as controlled by the computer, thus producing a passive right index movement. In the sequence condition, the left index finger was pushed down after a random period, while the right index finger was pushed down after 250 milliseconds. During the agency condition, the participant chose freely a time to push the left button down with her/his left index finger, while, as above, the right button and right index were passively pushed down under computer control 250 milliseconds later. In the remaining trials (i.e. other condition), an experimenter pushed the left button when she/he decided, thus triggering the participant's right button and index descent 250 milliseconds later. For all trials, judgment errors were the difference between perceived and actual time of occurrence of the beginning of the fall of the participant's right index finger. Patients with SCZ displayed an increased IB effect in the sequence, agency, and other conditions, suggesting that a previous event in the left hand was followed by an anticipation of the subjective time of another somatic sensory event, like a passive movement. Indeed, no between‐groups differences were found for baseline trials.

Voss et al.^42^ assessed IB in 24 patients with SCZ and 24 HCs with an experimental procedure implying a modified version of the Haggard's et al.^6^ method. In this study, the probability of the action causing the tone was manipulated.^42^ There were 32 trials each for baseline (voluntary actions not followed by tone) and the two experimental conditions (75% of actions, or 50% of actions followed by tones). To isolate the predictive contribution on IB, authors subtracted the average shift in the perceived time of action in ‘action only’ trials in the 50% effect probability condition, from the average time shift for actions on ‘action only’ trials in the higher predictability 75% effect probability condition. By focusing uniquely on ‘action only’ trials authors aimed at removing the retrospective influence of the tone (i.e. sensory effect) on action awareness. Conversely, the contribution of retrospective processes was calculated by subtracting the average shift in perceived time of action in ‘action only’ trials in the 50% effect probability condition from the average temporal shift for actions in ‘action and tone’ trials in the same condition. By focusing uniquely on the 50% condition, where predictability is lower, authors reduced the influence of predictability on this estimate. Patients with SCZ displayed, relative to HCs, a greater IB effect in each experimental condition. Furthermore, authors hypothesized a stronger retrospective contribution on action awareness in the SCZ group given that binding of actions towards tones in patients did not vary between the 50 and 75% tone frequency conditions, differently from HCs, who showed higher binding in the higher effect probability condition. In addition, an inverse correlation has been observed between positive symptom magnitude and prediction‐dependent shifts in action awareness.

Graham‐Schmidt et al.^47^ evaluated IB in 39 patients with SCZ (15 with passivity symptoms) and 43 HCs using an IE procedure. Participants were asked to judge the time interval between a computer keyboard button press and a subsequent stimulus. Time IEs were given across three different experimental conditions, i.e. in the ‘active’ condition, in which participants put one finger on the spacebar and intentionally pushed it, in the ‘passive’ condition, where the experimenter controlled the movement of the spacebar through an invisible wire that was connected to the spacebar. In the ‘other’ condition, an experimenter put her/his hand on the spacebar and pushed it intentionally. The intervals between button press and tone onset varied from 200 ms, to 400 or 600 ms. A significant time interval effect was observed in all three groups, with longer intervals being associated with increased perceived time intervals. Interaction contrasts showed that the increase in perceived interval with longer intervals significantly differed between HCs and patients with passivity symptoms, between HCs and patients without passivity symptoms, but not between subgroups with and without passivity. According to the investigators, these results may suggest that with increasing intervals, individuals with SCZ, independently from their passivity symptom profile, perceived the interval as being shorter than the one perceived by controls. Moreover, the authors analyzed the perceived interval, comparing the ‘active’ to the ‘passive’ condition and each of them to the ‘other’ for each of the 200, 400 and 600 ms intervals in the three groups. When collapsing time judgments across the delays, perceived interval was longer in the ‘active’ than in the ‘other’ condition only in HCs, but not in patients with SCZ, independently from whether they displayed passivity symptoms or not. In contrast, patients with passivity symptoms, compared to HCs and to patients without passivity symptoms, showed no significant differences in IE across all experimental conditions and all intervals. These results suggest that the modulation of time perception by voluntary movements may be impaired in SCZ according to specific symptoms of the patients.

To test the contribution of predictive and retrospective processes to IB effect in subjects at risk of developing a first episode of psychosis, Hauser et al.^43^ replicated the experimental design of the study of Voss et al.^42^ in 30 individuals with PP and compared them to 30 HCs. Although IB did not differ between the two groups, a stronger predictive contribution to IB in 75% tone frequency conditions was observed among individuals with PP, since predictive‐dependent shifts were greater in PP individuals, compared to HCs, while retrospective‐dependent shifts did not differ between PP individuals and HCs. Moreover, specific ego‐psychopathology symptoms, as assessed through Scharfetters^66^ Ego‐Psychopathology Scale, including consistency, demarcation, overcompensation, thought disorder, and bodily experience, were significantly correlated with predictive‐dependent shifts.

### Borderline personality disorder

BPD is characterized by a marked instability in emotional experiences, interpersonal relationships, self‐image, and impulsive behaviors.^67^ Similar to patients with SCZ, those with BPD display a weakened sense of self that may manifest through aberrant SoA and Sense of Ownership (SoO) (i.e. the experience of ‘mineness’ toward one's body).^68^ From a phenomenological perspective, disrupted agency in BPD is evident in a pattern where impulses are promptly acted upon so swiftly that the self is not experienced as the initiator of the behavior. When facing negative emotions, individuals with BPD often feel incapable of controlling or explaining some maladaptive behaviors, including self‐harm or binge eating.^69^, ^70^ Research has highlighted that alterations in specific brain regions, such as DLPFC and anterior CC, along with deficits in executive functions, including planning and decision‐making abilities, may account for reduced impulse control in individuals with BPD and exacerbate challenges in evaluating the repercussions of one's actions.^71^

#### IB studies in BPD

To test the interplay between SoO and SoA in BPD, Möller et al.^50^ applied to 21 patients with BPD and 21 HCs an experimental design that consisted of an IE procedure incorporated into an active rubber hand illusion (aRHI) task. Participants had their right hand placed on a lower button, right below an artificial hand that was placed on an upper button. The artificial index finger and the lower button where the participant had placed her/his right hand were connected with a string. Whenever the experimenter moved the artificial hand up or down (no agency condition), the index finger of the artificial hand followed these movements by going up or down, whereas when it was the participant to press the lower button (self‐agency condition), the index finger of the artificial hand moved down. Two further experimental conditions were adopted, i.e. a spatially congruent and a spatially incongruent condition. In the former, the artificial hand and the participant's hand were aligned, whereas in the latter, the artificial hand was misaligned with the participant's hand. Participants received the instruction to estimate the time interval from the beginning of button pressing and the perception of a subsequent tone, provided with different delays (100, 400 or 700 ms). In the self‐agency condition, participants executed the artificial index finger movements themselves, while in the no‐agency condition they saw, touched, and felt the artificial hand's index finger moving without their voluntary contribution. No significant differences were found between BPD patients and HCs in any of the experimental conditions.

### Autism spectrum disorders

ASDs are neurodevelopmental conditions that have a profound impact on social interaction, communication, and multiple behavioral and cognitive functions.^72^ While ASDs exhibit significant phenotypic diversity, a shared characteristic among most individuals with ASDs is a deficiency in self‐referential processing.^73^ These individuals often face specific challenges in discerning between their own mental states, actions, and emotions compared to those of others.^74^ At an implicit level, self‐referential processing involves bodily‐grounded mechanisms, which are based on proprioceptive and motor‐related signals, including SoA and SoO.^75^ Consistently with this conceptual framework, impaired action planning, and monitoring are commonly reported in individuals with ASDs.^76^ Previous studies in individuals with ASDs also reported dysfunctional pre‐SMA activity during action planning as well as altered activity in the dorsal CC with tasks requiring response monitoring.^77^ As previously mentioned, both these brain regions play a pivotal role in action prediction and SoA.

#### IB studies in ASDs

Two studies evaluated IB in ASDs. Sperduti et al.^45^ assessed 15 subjects with an ASD compared to 17 HCs through an IE procedure. In the operant condition, participants were required to press the spacebar of a keyboard at a time of their choice. Three different types of sensory stimuli, i.e. visual, auditory, and multimodal, were adopted. The first involved, a red dot on a computer screen, the second a tone, and the third both stimuli simultaneously; stimuli were presented after a variable temporal delay (250, 450, or 650 ms). In the control condition the participant's action was replaced by a warning tone, which was presented at fixed 1.5 s intervals after the start of the trial that was followed by the above‐mentioned visual, auditory, or multimodal stimuli. A proportion difference score was calculated for each interval and each modality by subtracting the participant's time estimation in the operant condition from the one in the control condition and dividing this difference by the physical actual duration of the interval to be estimated, so that the more positive the proportion difference score was, the greater the IB effect. A significant group effect in HCs compared to individuals with ASD was observed, resulting from a greater proportion difference score in the former. Besides this, the authors reported a significant main effect of modality and interval duration with significantly greater IB effect for 450 ms and 650 ms visual stimuli in HCs compared to individuals with ASD.

Finnemann and colleagues replicated Voss' et al.^42^ experimental procedure in 23 individuals with an ASD matched with 17 HCs.^52^ No significant differences between the two groups were observed, with both participants with ASD and HCs displaying increased IB in the 75% effect probability conditions compared to the 50% effect probability condition.

### Anorexia nervosa

AN is an eating disorder characterized by intense fear of gaining weight as well as by body image disturbances. Specifically, individuals with AN often exhibit obsessive thoughts about food, weight, and body image.^78^, ^79^ Multiple neurobiological hypotheses have been proposed to explain the disorder and neuroimaging studies have revealed structural alterations in the insula, a brain region associated with both body perception and action awareness.^80^ Albeit not listed among formal diagnostic criteria, the need for control is a core psychopathological feature of AN that also plays a central role in the initiation and persistence of the disorder.^81^ Consistently with some theories, dietary restrictions may be conceived of as attempts to compensate for deficits in the structure of the Self, including sense of bodily presence and self‐efficacy, possibly suggesting that the lack of control experienced by AN patients may be associated with impaired SoA.^32^, ^82^

#### IB studies in AN

Engel et al.^53^ explored SoA in 52 women with AN, of which 22 had active disorder and 30 had recovered from AN, and 29 age‐matched HCs through the method of Haggard et al.^6^ Overall binding did not differ among the three groups. However, explicit sense of control, measured through the Self‐Control Scale, was inversely correlated to tone binding in patients recovered from AN.

### Functional movement disorders

FMDs encompass a range of motor symptoms characterized by abnormal movements or postures that have no apparent organic or structural neurological basis.^83^ Although the exact etiology of these disorders remains uncertain, they are often associated with psychosocial, emotional, or psychiatric factors.^84^ Neuroimaging studies highlighted functional alterations in FMDs, including hypoactivation of the pre‐SMA and abnormal connectivity between the pre‐SMA and limbic areas.^85^ Attention shapes functional symptoms, leading to a remarkable reduction in their intensity when patients do not specifically concentrate on them. Conversely, during examinations, abnormal movements are frequently observed and can exhibit significant strength.^86^ Individuals with functional motor symptoms usually report abnormal action awareness^87^; studies involving action recognition and sensory attenuation tasks confirmed reduced SoA in FMDs.^88^, ^89^

#### IB studies in FMD

A single study investigated IB in 20 patients with FMDs compared to 20 age‐ and gender‐matched HCs.^44^ To test the effect of emotional response on action awareness in FMDs, binding scores were obtained through a computerized version of the LC after participants completed a conditioning phase where three different auditory tones were paired with positive, negative or neutral emotional stimuli. Patients displayed significantly lower tone and overall binding scores compared to HCs. However, no significant effect was observed for emotional valence on binding, in either HCs or patients with FMDs.

### Parkinson's disease

PD is a neurodegenerative disorder involving motor function alterations, including bradykinesia, tremor, and postural difficulties. These symptoms are primarily caused by changes in dopamine release in the substantia nigra.^90^ Therefore, dopaminergic medications are considered the standard treatment. PD may affect the perception and awareness of voluntary action.^91^ For example, the perception of the position and motion of one's own body parts (i.e. kinaesthesia) is impaired in PD,^92^ possibly due to abnormal sensory feedback processing.^93^ Given the difficulty in initiating and controlling voluntary movements, SoA disturbances in PD patients are expected.^89^

Impulsive‐compulsive behaviors (ICBs) are common neuropsychiatric complications associated with dopaminergic medications and typically involve an impaired subjective experience of volition associated with action.^94^ The impact of dopaminergic medication on SoA is also apparent based on sensory attenuation studies conducted in PD. The degree of attenuation of self‐generated stimuli in individuals with PD is negatively related to the severity of motor symptoms and positively related to individual dopamine dosing, thus supporting the hypothesis that voluntary movements abnormalities in PD partly depend on impaired sensorimotor integration.^95^

#### IB studies in PD

Three different studies evaluated IB in patients with PD. Moore et al.^21^ tested nine patients with PD, both on‐ and off‐dopaminergic medication and compared them to nine age‐matched HCs. Temporal binding was explored through the method of Haggard et al.^6^ Overall binding did not significantly differ between off‐medication PD patients and HCs, possibly indicating that abnormal experience of agency is not a feature of PD *per se*. However, overall binding in patients with PD on dopaminergic medication was significantly greater than in off‐medication PD patients.

Ricciardi et al.^49^ replicated the classic experimental procedure of Haggard et al.^6^ in 19 PD patients with ICBs, 19 PD patients without ICBs, and 19 HCs matched for age, gender, and educational level. PD patients with ICBs displayed a significantly greater action binding than HCs and patients without ICBs, which indicates an abnormally large shift in perceived timing of the action towards the tone. No difference regarding action binding was observed between patients without ICBs and HCs. Similarly, tone binding and overall binding did not differ among the three groups.

Saito et al.^48^ investigated IB through the classic LC procedure in 19 patients with PD on regular dopaminergic medication ad 19 HCs matched for age, gender and education level. To test whether aberrant action awareness in PD are related to deficits in primary components of central sensorimotor processing or to secondary effects of motor impairment, patients with PD performed the task with both hands, i.e. of the more and of the less affected side by motor symptoms. Relative to HCs, patients with PD displayed significantly decreased action binding, whereas no significant differences between the two groups were observed for tone binding. No significant effect of hand was detected in both operant conditions. The authors suggest that patients with PD experience a change of action awareness at an implicit level, with strong involvement of sensorimotor processing.

### Cortico‐basal syndrome

CBS is a progressive movement disorder distinguished by asymmetrical cortical and extrapyramidal dysfunctions.^96^ CBS can manifest without a discernible biological cause, although it is typically attributed to underlying neurodegenerative conditions.^97^ CBS is commonly linked to two volitional action disorders, alien limb and apraxia. Alien limb refers to the execution of semi‐purposeful movements without conscious intention, while apraxia involves difficulties in performing complex movements despite understanding their goal.^89^ Multiple studies provide evidence that individuals with alien limb syndrome and those with apraxia commonly have disturbed SoA.^98^, ^99^, ^100^, ^101^ Neuroimaging findings in individuals with CBS reveal atrophy of peri‐rolandic regions, including the primary motor cortex, along with variable involvement of the inferior parietal cortex, left SMA, and basal ganglia. Additionally, abnormalities in white matter tracts connecting the frontal and parietal lobes have been documented^97^). Notably, widespread hypometabolism in the frontal, temporal, and parietal lobes is often observed in CBS, particularly on the opposite side of the body that is most affected by motor deficits, such as alien limb and apraxia.^102^

#### IB studies in CBS

Wolpe et al.^46^ assessed 10 patients with CBS compared to 16 age‐ and sex‐matched HCs. The experimental procedure of Haggard et al.^6^ was adopted to isolate the contribution of action shifts and tone shifts to IB. As CBS typically presents with an asymmetrical motor pattern, after completing the task with one hand, participants performed it with the other. In HCs perception of action and tone did not differ between hands, whereas patients displayed a significantly increased action binding when the task was performed with the more‐affected hand compared to the less‐affected one. Across groups, action binding was increased in the more‐affected hand compared to HCs, whereas in the less‐affected hand it did not differ from controls. Moreover, action binding in the more‐affected hand positively correlated with both the number of reported alien limb symptoms in that hand and with the severity of apraxia. In a separate experimental session, all HCs and eight patients underwent a magnetic resonance imaging scan. Action binding in the most‐affected hand significantly correlated with gray matter volume in the pre‐SMA among patients with CBS. Moreover, it also correlated with white matter deficit in several areas, including white matter adjacent to the pre‐SMA and PFC, the anterior corpus callosum, and the superior longitudinal fasciculus, as well as with functional connectivity between the pre‐SMA and bilateral DLPFC, dorsal CC, cerebellum, and intraparietal sulcus.

### Gilles de la Tourette syndrome

GTS is a movement disorder that typically emerges during childhood and is characterized by hyperkinetic movements and abnormal vocalizations, known as tics. The severity and occurrence of tics can be influenced by various environmental factors. Stress and anxiety are commonly known triggers that induce and intensify tics. Conversely, when individuals with GTS are relaxed or engaged in activities requiring concentration or physical effort, the severity and frequency of tics are reduced.^103^ Many GTS patients report experiencing ‘premonitory urges’ that precede their tics. These urges are characterized by uncomfortable sensory sensations such as restlessness, pain, pressure, mounting tension, or vague discomfort. The only relief from these sensations comes from expressing the tic.^104^ The semi‐voluntary nature of tics may be linked to an abnormal conscious experience of action^105^ and the capacity to inhibit tics based on the conscious awareness of the intention to move.^106^ However, the decision to carry out the tic is typically perceived as a voluntary response to alleviate the unpleasant sensation.^105^ Individuals with GTS exhibit various changes in brain structure and functional connectivity.^107^ In particular, Hampson et al.^108^ observed impaired temporal cross‐correlation of the motor cortex and the SMA in individuals with GTS, including abnormally elevated SMA activity, in the seconds preceding and following tic expression.

#### IB studies in GTS

Zapparoli et al.^51^ explored IB in 25 patients with GTS compared to 25 age‐ and gender‐matched HCs through an IE procedure. During the execution of the task fMRI scans were performed. In operant conditions, participants were instructed to press a button with their right index finger on a key‐pad placed under their right hand. Upon pressing the button, a light‐bulb on a computer screen was presented with a variable delay of 200, 400, or 600 ms. Time judgments were reported through a visual analog scale to which participants responded through a 5‐key‐pad placed under their left hand. In the passive condition, an experimenter pressed participants' right index fingers to turn the light‐bulb on, while participants stayed still. Participants were then asked to compute the action‐outcome delay the same way they did for active trials. Behavioral results highlighted that perceived time compression was significantly stronger in operant compared to passive conditions only in HCs and only for 200 ms‐delayed stimuli. At longer delays, the temporal compression did not differ between operant and control conditions in both groups. Furthermore, a significant correlation between the temporal compression values recorded during the active condition at 200 ms and the severity of motor symptoms was observed in patients with GTS. Higher negative values of temporal compression (i.e. estimated time interval shorter than the real one) at the 200 ms action‐outcome delay corresponded to higher BOLD activity in several brain areas for the active trials compared to passive ones in HCs, including left pre‐SMA, bilateral superior frontal gyrus, left precentral gyrus, postcentral gyrus, superior parietal lobule, insular cortex, bilateral cerebellum, and left hippocampus. Conversely, no brain region significantly correlated with IB magnitude in patients with GTS.

## Discussion

To our knowledge, the current review is the first to systematically target the IB effect in several neuropsychiatric disorders, including SCZ, BPD, ASD, FMDs, PD, GTS, AN, and CBS. Fifteen studies involving a total of 357 patients with a psychiatric or neurological diagnosis were included in this review. Overall, most included studies showed differences in behavioral performance on tasks between patients and HCs which show‐off as either increased or diminished IB, based on the disorder that was explored.

The ‘hyper‐binding’ effect observed across most included studies in individuals with SCZ^40^, ^41^, ^42^, ^47^ suggests an implicit over‐attribution of sensory consequences of movement to oneself in this population which may ultimately lead to abnormal SoA.^4^ This hyper‐binding effect is also consistent with findings of studies exploring SoA in SCZ with different experimental paradigms, which highlight a tendency for patients to hyper‐associate their actions and outcome.^62^, ^109^, ^110^ Importantly, abnormalities in the temporal context of sensory processing in SCZ may not only affect the integration of external inputs, such as auditory and visual information from the environment,^111^ but also body‐related somatosensory stimuli.^112^ Accordingly, an increased experience of causality or feeling of agency arising from a stronger IB effect may result in the delusional belief of being in control of other people's thoughts and actions (or being controlled by them), or to control external events in the environment that one could not feasibly or credibly influence.^113^ However, from a psychopathological perspective, unusual experience of agency in SCZ typically involve decreased, rather than increased, feeling of control of one's own volitional actions.^114^ Passivity symptoms, where individuals with SCZ regard some of their movements as not being the result of their own will and, usually, as being controlled by outside forces, are a paradigmatic example. Of note, as suggested by Graham‐Schmidt et al.,^47^ patients with passivity symptoms may not display action‐modulation of time perception by voluntary movements as opposed to SCZ patients without passivity symptoms, who discriminate between active and passive movements but perceive consequences of other people's actions as if they were performing them themselves. Taken together, these findings highlight the complex relationship between putative measures of SoA, including IB, and subjective experiences of agency in SCZ. There is evidence that abnormal action‐outcome binding in SCZ may depend upon specific deficits of predictive anticipation of external effects of one's own action, as indicated by Voss et al.^42^ What the latter study suggested was that action binding in individuals with SCZ may not vary with outcome predictability but is rather strongly modified by the actual occurrence of the outcome itself, suggesting a strong retrospective influence. Accordingly, SoA in SCZ appears to be driven mostly by sensory external cues, given unreliable internal sensorimotor predictions specifying the prior probability of an outcome, given an action. These finding are consistent with neurocognitive theories emphasizing the role of impaired sensorimotor predictions in generating positive symptoms in SCZ.^115^ Remarkably, this pattern of predictive deficits in IB may change during the progression of schizophrenic illness. As highlighted by Hauser et al.,^43^ individuals with PP, unlike patients with full‐blown SCZ, may display enhanced predictive contributions to SoA, as measured by IB, rather than abnormalities in the magnitude of the effect *per se*. The finding of hyper‐prediction in the psychotic prodromes and hypo‐prediction in established schizophrenic illness is consistent with studies highlighting neurochemical changes associated with disease progression. In the prodromal stage of SCZ, enhanced error signaling, sustained by increased glutamatergic transmission in the PFC, may strengthen causal associations between actions and outcomes leading to stronger predictions.^116^, ^117^ Subsequent dopaminergic neurotransmission dysfunction in the later stages of SCZ may instead affect glutamatergic‐driven error signaling by introducing noise to that signal.^118^ Accordingly, the alteration of prediction error signaling by abnormal dopaminergic activity would explain the transition from excessive, yet valid internal sensorimotor predictions in patients experiencing initial symptoms of psychosis to unreliable predictions, as observed in patients with established SCZ.

The contribution of aberrant dopamine transmission to abnormal SoA experience is corroborated by studies investigating IB in patients with PD, both on‐ and off‐dopaminergic drug treatment.^22^, ^48^, ^49^ Taken together, these studies suggest that PD itself may not be associated with impaired SoA but rather, that a subgroup of patients with PD, including those taking regular dopaminergic medications and reporting ICBs, may display abnormally increased action‐outcome binding. This is of particular interest, as ICBs may be a relatively common side‐effect in patients taking dopamine agonists for PD and involve a weakened subjective experience of volition associated with actions.^94^ Previous studies have examined the role of dopamine in modulating motor cognition in PD, with administration of dopaminergic medication alleviating some cognitive functions, while simultaneously impairing other.^119^ According to the overdose theory, the differential impact of dopaminergic medication on cognitive function is linked to the particular pattern of dopamine depletion that takes place as PD progresses. In the initial stage of the disease, dopamine depletion is higher in the dorsal compared to the ventral striatum. As a result, dopaminergic medication has been shown to enhance cognitive functions that rely on the dorsal striatum, while having a negative impact on cognitive functions supported by the ventral striatum.^120^ The ventral striatum is crucial in instrumental learning and performance,^121^ including reinforcement of action‐effect association that contributes to the IB effect.^33^ Dopaminergic medications affect striatal reward prediction error signals, which play a key role in causal learning, boosting formation of associations between actions and reward outcomes in healthy volunteers.^122^ In line with this conceptual framework, dopaminergic medication also proved to modulate the weight given to internal predictive signals versus external sensory cues in PD, with PD patients on‐medication relying more on current sensory information compared to prior information in decision‐making under uncertainty.^123^ According to available evidence, ICBs are associated with excessive stimulation of dopamine receptors in the limbic striatum,^124^ which contributes to the development of these disorders by promoting the formation of instrumental action‐effect associations through enhanced learning^125^ and incentive signaling.^126^ Taken together, this evidence suggests that increased SoA, sustained by abnormal dopaminergic activity in the sensorimotor systems which compute prediction errors,^127^ may play a role in the development of ICBs. Accordingly, perceiving one's actions as highly effective can lead to a propensity to engage in actions that would typically be restrained, as in the case of patients with PD reporting ICBs.^22^

Impaired awareness of volitional action, which may involve both abnormal movements in the absence of volition as well as the inability to perform purposeful actions, despite recognizing the goal of the action, may be a distinguishable feature shared by a number of neuropsychiatric disorders, including, CBS, GTS, and FMD. Of note, included studies that explored SoA in these disorders^44^, ^46^, ^51^ found a significant group effect on the magnitude of IB, with individuals with GTS and those with FMDs showing decreased binding of the perceived time of actions towards their effects, and individuals with CBS reporting increased action‐outcome binding for the most affected hand, relative to HCs. Contrary to PD, where external cues, including object affordance, can improve motor performance,^128^ involuntary movements in FMDs are rarely influenced by environmental factors but, instead, vary according to psychological, internal factors, and attention.^86^ Stenner and Haggard^129^ highlight the significance of ‘precipitating physical events’ in understanding the greater reliance on internal cues rather than external cues in the behavior of patients with FMDs. They suggest that events like physical injuries or panic attacks, which serve as ‘precipitating events,’ are subjectively interpreted by FMDs patients as indicative of a ‘loss of control’, ultimately leading to increased monitoring of actions. This increased monitoring would be accompanied by expectations or predictions of a robust and vivid sense of being in control during movement, which collide with the physiological ‘thin’ conscious experience of control that the motor system normally provides over actions. This mismatch between expected and actual conscious experience of control during actions would be interpreted by patients with FMDs as abnormal. The increased focus on motoric details of the action, including parameters of motor execution, would also lead to a more precise perception of the sensory outcomes of movement which may ultimately lead to decreased tone and overall binding as observed by Kranick et al.^44^

In both patients with CBS and patients with GTS, abnormal action‐outcome binding significantly correlated both with the severity of the diseases in terms of motoric symptoms, as well as with functional changes in the activity of a fronto‐parietal network with its hub in the pre‐SMA. pre‐SMA activity supports voluntary behavior, including the intention‐to‐act experience^130^ and action decisions in the absence of external or learned cues.^131^ There is evidence that disruption in this area is linked to abnormal action‐effect binding.^132^

The increased action binding found among individuals with CBS by Wolpe et al.^46^ may depend upon abnormal integration of agency cues. According to this conceptual framework, when there is low reliability or high uncertainty in perceiving the timing of one's own actions, individuals may overly rely on the sensory feedback to perceive their own actions.^100^ This interpretation is supported by the finding that the accuracy of time estimation in baseline conditions was correlated with action binding, as predicted by cue integration theory for IB^12^ The authors hypothesized that the reduced precision in volitional signals that drive internally generated actions (and inhibit actions triggered by the environment) may be a result of gray matter degeneration in the pre‐SMA and in white matter tract integrity of its connections with PFC.^46^

We may interpret the decreased IB effect observed in patients with GTS as indicative of abnormal monitoring of action consequences. GTS patients may struggle with updating forward model estimates, which are crucial for adjusting ongoing motor plans based on the sensory consequences generated by their actions.^133^ This difficulty in updating forward models may explain why GTS patients do not exhibit the IB effect between their actions and the resulting sensory effects in the external environment. In contrast to what was observed in HCs, individuals with GTS also did not exhibit any noticeable correlation between the activity of the pre‐SMA and the magnitude of the IB effect.^51^ This finding, coupled with neurophysiological evidence of abnormal excitatory activity from the SMA to the putamen,^134^ and reduced striatal inhibition in various aspects of tic generation and suppression,^135^ and delayed awareness of the intention to voluntarily generate or suppress movement (i.e. to tic or not to tic),^136^ point at a failure of voluntary control in GTS.

Studies addressing IB in ASD provided diverging results, with one study^45^ finding an attenuated action‐effect binding in adults with autism when tested with visual, auditory and audio‐visual action outcomes, while another study^52^ found no significant differences between individuals with ASD and controls in the magnitude of temporal attraction of voluntary actions and their outcomes. However, the two studies were heterogeneous both in terms of the task employed to assess IB (LC method with different probability conditions versus IE method with visual, auditory and audio‐visual stimuli) and in terms of sample (male‐only participants in the Sperduti and colleagues experiment [2014]). Accordingly, no definitive conclusion can be drawn. Finally, two single studies, namely Möller et al.^50^ and Engel et al.^53^ failed to detect significant differences in implicit SoA among individuals with BPD and AN, respectively, suggesting that the specific mechanisms underlying the IB task are not impaired or altered in these disorders.

Surprisingly, no studies assessed implicit measures of SoA in mood disorders, despite in recent years increasing attention has been directed to the involvement of affective states in the process of feeling of agency.^1^ Interestingly, there is evidence for the modulation of SoA by affective significance of action outcomes, with IB that is attenuated for negative compared to positive or neutral outcomes. Importantly, these specific effects of emotional valence on SoA seem to be automatic and implicit, thus suggesting low‐level sensorimotor processes to be involved.^137^, ^138^ Intriguingly, a study by Obhi et al.^139^ reported that action‐effect interval estimates are significantly longer in healthy volunteers when they are instructed to remember a depression‐inducing event than after remembering non‐specific events, like those that occurred the previous day, or in a baseline condition. A more recent study showed that deprivation of personal control can lead to learned helplessness and decrease the IB effect^140^ while depressive traits were linked to a limitation in the variability of IB between free and forced conditions in a classical LC paradigm.^141^ Taken together, the above evidence points toward a role of emotional experience in shaping SoA, including psychopathology of mood.

Although definitive conclusions cannot be drawn, taken together, results indicate that disrupted SoA in neuropsychiatric disorders can manifest in different ways, encompassing both ends of the spectrum, including either over‐ or under‐attribution of the SoA to the self. We may attribute the increased IB effect in SCZ, PD, and CBS to an over‐reliance on external sensory action events, which is, in turn, partly due to an under‐reliance on internal action cues or sensorimotor predictions. Conversely, patients with FMDs and GTS may be excessively reliant upon internal, pathological predictions concerning movement. This can be attributed to challenges in updating forward dynamic predictions based on the sensory consequences produced by their actions.^142^ In line with an optimal cue integration framework, under typical circumstances, the perception of being the initiator of one's own actions emerges from a dynamic interplay among various factors. This includes prior expectations, internal predictions regarding the sensory outcomes of actions, incoming sensory information, and post‐hoc beliefs. These cues are not mutually exclusive but are combined and weighted based on their relative reliability to establish a robust representation of agency in a particular situation.^143^ Accordingly, SoA misattribution, both in terms of over‐attribution and under‐attribution, may stem from a context‐dependent and weighted integration of imprecise internal predictions alongside alternative agency cues.^144^ Importantly, several brain structures which are thought to underlie SoA abnormalities in neuropsychiatric disorders, including the DLPFC, the pre‐SMA and the premotor cortex, the PPC, as well as the precuneus normally;^37^ serve as neural hubs for multimodal integration of sensorimotor information within motor control networks.^145^ Some issues that may limit the generalizability of our results must be acknowledged. First, the number of eligible studies addressing IB in neuropsychiatric disorders was relatively low. Some disorders, like SCZ and PD were investigated by multiple studies, while for most other neuropsychiatric conditions, only one study was found for each of them. Furthermore, the methodologies adopted for studying the different disorders were heterogeneous. Indeed, although most studies used a classic LC paradigm, in some cases this was modified, thus adding methodological heterogeneity. It is important to acknowledge that there are unresolved issues and limitations associated with the paradigm of IB that we should carefully consider. According to some lines of evidence, temporal binding, as observed in IB, is not exclusive to one's own actions. It can also occur when observing the actions of another individual^146^ or even when observing a predicted action‐effect sequence generated by a machine.^147^ These findings indicate that IB might simply reflect the general temporal binding that arises from learning, the causal relationships between actions, and their effects. However, as suggested by other lines of evidence,^148^, ^149^ both intentionality and causality need to be present simultaneously for the occurrence of IB.^4^ Further, most IB experiments have been carried out in situations where individuals perform actions on their own. However, in typical daily scenarios, people rarely act in isolation; instead, they engage in social contexts and often participate in ‘joint actions" with others. Based on their joint action experiments, Obhi and Hall^150^ proposed that when individuals are paired with another person, a new collective identity of ‘we’ is automatically established. In this context, both individuals experience a sense of shared agency or pre‐reflective agency, as evidenced by IB, for any action performed by either partner. However, at a more reflective level of processing, they still believe that only one person is responsible for causing the tone. Further studies are therefore warranted to explore SoA and IB in joint human actions, especially among individuals with neuropsychiatric disorders. These concerns underscore the significance of investigating the underlying mechanisms involved in the phenomenon of binding. Despite the potential limitations of the paradigm, IB still offers a valuable means to objectively quantify fundamental aspects of SoA in both health and disease. Future studies could benefit from the combination of pharmacological challenges with neuroimaging techniques so to elucidate some neurobiological aspects of SoA in neuropsychiatric disease. For example, ketamine, a compound that shows both psychotomimetic and antidepressant properties,^151^ was found to boost agentic experience in healthy individuals.^152^ Besides, evidence coming from both preclinical and clinical studies highlight that ketamine may hold potential in ameliorating motor symptoms in disorders involving impaired awareness of volitional action, including TS, PD, and FMD,^153^, ^154^, ^155^ possibly via the functional restoration of neural circuitries that sustain normal SoA. Similarly, non‐invasive stimulation methods of selected areas, such as transcranial direct current stimulation (tDCS) over DLPFC, affect the temporal binding of actions towards tones in studies involving endogenous action selection. Of note, tDCS on DLPFC proved to be effective in a number of neuropsychiatric conditions, including SCZ,^156^ depression,^157^, ^158^ and PD,^159^, ^160^ which share the subjective experience of impaired control over one's own actions. In conclusion, a deeper understanding of the role of SoA in neuropsychiatric diseases, including neuromodulatory failure sustaining agency misattribution, could provide novel insight into the study of the pathophysiology of these disorders as well as a quantitative view of psychopathology that may enrich traditional psychiatric classification^161^ and contribute to tailor therapeutic interventions.

## Author contributions

Conception/design of the study: L.M, M.D.L, G.D.K; data analysis; P.L, M.M; drafting manuscript and figures/tables: L.M, D.J, M.D.N, E.C, M.D.L; review/editing, L.M, G.D.K, V.G, M.A, M.A. (Marianna Ambrosecchia); supervision V.G., L.J., G.S.

## Disclosure statement

The authors declare no conflict of interest.

## Supporting information


**DATA S1** Supporting Information.
