# Supplementary material for: Sense of agency and its disturbances: A systematic review targeting the intentional binding effect in neuropsychiatric disorders
Source: Psychiatry Clin Neurosci. 2023 Nov 28;78(1):3–18. doi: 10.1111/pcn.13601 (PMC11488622; doi:10.1111/pcn.13601)
Supplement: Supplementary file 1 — DATA S1 Supporting Information. [file PCN-78-3-s001.docx]

**Supporting Information**

*Search Strategy*

We conducted a systematic research on the PubMed/MEDLINE database from inception to 18-November-2022, using “intentional binding” as search strategy. We also searched the ClinicalTrials.gov site on the same date to identify whether current trials investigating “intentional binding” had data on neuropsychiatric patients. Authors agreed on search strategy and eligibility criteria, which were: (1) original research studies; (2) studies including patients with a neurological or a psychiatric diagnosis; diagnoses ought to be based on the Structured Clinical Interview for DSM-IV/5, or on other commonly accepted clinical diagnostic criteria worldwide, including commonly used diagnostic interviews or the ICD-9/11; (3) studies using tasks that calculate the IB effect; (4) studies providing data separately for healthy controls (HCs), if included, and for each single neuropsychiatric diagnosis.

Exclusion criteria were: (1) studies assessing HCs only; (2) studies not assessing the IB effect through a specific task (labelled No IB); (3) reviews and meta-analyses, cumulatively labelled as Reviews (however, their reference lists were hand-searched to seek additional eligible studies that could possibly have eluded our search strategy); (4) letters to the editor, editorials, hypotheses, conceptual articles, and opinion papers not supported by authors’ original data, which were collectively labelled as Opinions; (5) serendipitous/accidental search result with no actual relation to the subject matter (labelled as Unrelated); (6) studies not providing data separately for neuropsychiatric patients and HCs, but rather lumped the neuropsychiatric diagnoses and provided general data only (i.e., included patients with neuropsychiatric diagnoses, but did not provide data separately according to diagnosis), labelled as Lumping; (7) qualitative studies or those using estimates and tasks not actually tested (labelled as No data); (8) correction to existing article (labelled as Duplicate; however, we took into account corrections when dealing with the original study).

We run Delphi rounds to reach consensus on whether a study should be included or excluded. We adhered to the 2020 *P*referred *R*eporting *I*tems for *S*ystematic reviews and *M*eta-*A*nalyses (*PRISMA*) statement (Page et al., 2021). We extracted the data of eligible studies using a standardized spreadsheet. We recorded the following variables: name of authors or first author et al. if more than two, publication year, sample size, participants’ age, biological sex ratio (male/female), task design, results, and conclusions over the association between neuropsychiatric diagnosis and changes in IB effect. Results are shown in the PRISMA flowchart (**Figure S1**). Detailed information about each study regarding inclusion or reason for exclusion can be found in **Table S1**.

Risk of bias (RoB) of included studies was assessed using the Cochrane RoB 2.0 tool (Higgins et al., 2022) (**Table S2**).

**Figure S1.** PRISMA flowchart of our search strategy and selection of eligible studies.

**Identification of studies via databases and registers**

Records identified from:

-Databases (N = 1) n=198

-Registers (N = 1) n=2 (2 new)

-Records added from reviews’ reference lists (n = 8)

Records removed *before screening*:

-Duplicate records removed (n = 0)

-Records marked as ineligible by automation tools (n = 0)

-Records removed for other reasons (n = 2, unrelated to the subject matter)

**Identification**

Records screened

(n = 206)

Records excluded**

(n = 0); no automation tools were used

Reports sought for retrieval

(n = 206)

Reports not retrieved

(n = 0)

**Screening**

Reports excluded (N=191):

-Healthy controls (n = 164)

-Reviews (n = 19)

-Opinions (n = 5)

-No data (n = 2)

-Did not use IB (n = 1)

Reports assessed for eligibility

(n = 206)

Studies included in review

(n = 15)

Reports of included studies

(n = 15)

**Included**

**Table S1.** Detailed information about each study regarding inclusion or reason for exclusion

| 1 | Haggard P, Clark S, Kalogeras J. Voluntary action and conscious awareness. Nat Neurosci. 2002;5(4):382-5. doi: 10.1038/nn827. | HCs |
| --- | --- | --- |
| 2 | Tsakiris M, Haggard P. Awareness of somatic events associated with a voluntary action. Exp Brain Res. 2003;149(4):439-46. doi: 10.1007/s00221-003-1386-8. Epub 2003 Feb 19. | HCs |
| 3 | Haggard P, Clark S. Intentional action: conscious experience and neural prediction. Conscious Cogn. 2003;12(4):695-707. doi: 10.1016/s1053-8100(03)00052-7. | HCs |
| 4 | Yarrow K, Johnson H, Haggard P, Rothwell JC. Consistent chronostasis effects across saccade categories imply a subcortical efferent trigger. J Cogn Neurosci. 2004;16(5):839-47. doi: 10.1162/089892904970780. | HCs |
| 5 | Liddle E, Jackson SR. Recalibrating time: when did I do that? Curr Biol. 2006;16(23):R994-6. doi: 10.1016/j.cub.2006.10.042. Epub Dec 5 2006. | Opinion |
| 6 | Engbert K, Wohlschläger A, Thomas R, Haggard P. Agency, subjective time, and other minds. J Exp Psychol Hum Percept Perform. 2007;33(6):1261-8. doi: 10.1037/0096-1523.33.6.1261. | HCs |
| 7 | Wenke D, Haggard P. How voluntary actions modulate time perception. Exp Brain Res. 2009;196(3):311-8. doi: 10.1007/s00221-009-1848-8. Epub 2009 May 27. | HCs |
| 8 | Haggard P, Poonian S, Walsh E. Representing the consequences of intentionally inhibited actions. Brain Res. 2009;1286:106-13. doi: 10.1016/j.brainres.2009.06.020. Epub 2009 Jun 12. | HCs |
| 9 | Buehner MJ, Humphreys GR. Causal binding of actions to their effects. Psychol Sci. 2009;20(10):1221-8. doi: 10.1111/j.1467-9280.2009.02435.x. Epub 2009 Sep 2. | HCs |
| 10 | Ebert JP, Wegner DM. Time warp: authorship shapes the perceived timing of actions and events. Conscious Cogn. 2010;19(1):481-9. doi: 10.1016/j.concog.2009.10.002. Epub 2009 Nov 6. | HCs |
| 11 | Moore JW, Haggard P. Intentional binding and higher order agency experience. Conscious Cogn. 2010;19(1):490-1. doi: 10.1016/j.concog.2009.11.007. Epub 2010 Jan 4. | Opinion |
| 12 | Strother L, House KA, Obhi SS. Subjective agency and awareness of shared actions. Conscious Cogn. 2010;19(1):12-20. doi: 10.1016/j.concog.2009.12.007. Epub 2010 Feb 1. | HCs |
| 13 | Cravo AM, Claessens PM, Baldo MV. The relation between action, predictability and temporal contiguity in temporal binding. Acta Psychol (Amst). 2011;136(1):157-66. doi: 10.1016/j.actpsy.2010.11.005. Epub 2010 Dec 24. | HCs |
| 14 | Sperduti M, Delaveau P, Fossati P, Nadel J. Different brain structures related to self- and external-agency attribution: a brief review and meta-analysis. Brain Struct Funct. 2011;216(2):151-7. doi: 10.1007/s00429-010-0298-1. Epub 2011 Jan 7. | Review |
| 15 | Morey CC. Maintaining binding in working memory: comparing the effects of intentional goals and incidental affordances. Conscious Cogn. 2011;20(3):920-7. doi: 10.1016/j.concog.2010.12.013. Epub 2011 Jan 26. | HCs |
| 16 | Moore JW, Turner DC, Corlett PR, Arana FS, Morgan HL, Absalom AR, Adapa R, de Wit S, Everitt JC, Gardner JM, Pigott JS, Haggard P, Fletcher PC. Ketamine administration in healthy volunteers reproduces aberrant agency experiences associated with schizophrenia. Cogn Neuropsychiatry. 2011;16(4):364-81. doi: 10.1080/13546805.2010.546074. Epub 2011 Feb 6. | HCs |
| 17 | Aarts H, van den Bos K. On the foundations of beliefs in free will: intentional binding and unconscious priming in self-agency. Psychol Sci. 2011;22(4):532-7. doi: 10.1177/0956797611399294. Epub 2011 Feb 11. | HCs |
| 18 | Scott PM. Recent research on fumonisins: a review. Food Addit Contam Part A Chem Anal Control Expo Risk Assess. 2012;29(2):242-8. doi: 10.1080/19440049.2010.546000. Epub 2011 Jul 12. | Unrelated |
| 19 | Desantis A, Roussel C, Waszak F. On the influence of causal beliefs on the feeling of agency. Conscious Cogn. 2011;20(4):1211-20. doi: 10.1016/j.concog.2011.02.012. Epub 2011 Mar 10. | HCs |
| 20 | Obhi SS, Hall P. Sense of agency and intentional binding in joint action. Exp Brain Res. 2011;211(3-4):655-62. doi: 10.1007/s00221-011-2675-2. Epub 2011 Apr 19. | HCs |
| 21 | Obhi SS, Hall P. Sense of agency in joint action: influence of human and computer co-actors. Exp Brain Res. 2011;211(3-4):663-70. doi: 10.1007/s00221-011-2662-7. Epub 2011 Apr 19. | HCs |
| 22 | Moretto G, Walsh E, Haggard P. Experience of agency and sense of responsibility. Conscious Cogn. 2011;20(4):1847-54. doi: 10.1016/j.concog.2011.08.014. Epub 2011 Sep 14. | HCs |
| 23 | Aarts H, Bijleveld E, Custers R, Dogge M, Deelder M, Schutter D, Haren NE. Positive priming and intentional binding: eye-blink rate predicts reward information effects on the sense of agency. Soc Neurosci. 2012;7(1):105-12. doi: 10.1080/17470919.2011.590602. Epub 2011 Sep 22. | HCs |
| 24 | Waszak F, Cardoso-Leite P, Hughes G. Action effect anticipation: neurophysiological basis and functional consequences. Neurosci Biobehav Rev. 2012;36(2):943-59. doi: 10.1016/j.neubiorev.2011.11.004. Epub 2011 Nov 17. | Review |
| 25 | Moore JW, Obhi SS. Intentional binding and the sense of agency: a review. Conscious Cogn. 2012;21(1):546-61. doi: 10.1016/j.concog.2011.12.002. Epub 2012 Jan 11. | Review |
| 26 | Desantis A, Hughes G, Waszak F. Intentional binding is driven by the mere presence of an action and not by motor prediction. PLoS One. 2012;7(1):e29557. doi: 10.1371/journal.pone.0029557. Epub 2012 Jan 17. | HCs |
| 27 | Berberian B, Sarrazin JC, Le Blaye P, Haggard P. Automation technology and sense of control: a window on human agency. PLoS One. 2012;7(3):e34075. doi: 10.1371/journal.pone.0034075. Epub 2012 Mar 30. | HCs |
| 28 | Hughes G, Desantis A, Waszak F. Mechanisms of intentional binding and sensory attenuation: the role of temporal prediction, temporal control, identity prediction, and motor prediction. Psychol Bull. 2013;139(1):133-51. doi: 10.1037/a0028566. Epub 2012 May 21. | Review |
| 29 | Nolden S, Haering C, Kiesel A. Assessing intentional binding with the method of constant stimuli. Conscious Cogn. 2012;21(3):1176-85. doi: 10.1016/j.concog.2012.05.003. Epub 2012 Jun 21. | HCs |
| 30 | Obhi SS, Swiderski KM, Brubacher SP. Induced power changes the sense of agency. Conscious Cogn. 2012;21(3):1547-50. doi: 10.1016/j.concog.2012.06.008. Epub 2012 Jul 9. | HCs |
| 31 | Moore JW, Cambridge VC, Morgan H, Giorlando F, Adapa R, Fletcher PC. Time, action and psychosis: using subjective time to investigate the effects of ketamine on sense of agency. Neuropsychologia. 2013;51(2):377-84. doi: 10.1016/j.neuropsychologia.2012.07.005. Epub 2012 Jul 17. | HCs |
| 32 | Haering C, Kiesel A. Mine is earlier than yours: Causal beliefs influence the perceived time of action effects. Front Psychol. 2012;3:393. doi: 10.3389/fpsyg.2012.00393. eCollection Oct 8 2012. | HCs |
| 33 | Kühn S, Brass M, Haggard P. Feeling in control: Neural correlates of experience of agency. Cortex. 2013;49(7):1935-42. doi: 10.1016/j.cortex.2012.09.002. Epub 2012 Sep 17. | HCs |
| 34 | Buehner MJ. Understanding the past, predicting the future: causation, not intentional action, is the root of temporal binding. Psychol Sci. 2012;23(12):1490-7. doi: 10.1177/0956797612444612. Epub 2012 Oct 25. | Opinion |
| 35 | Takahata K, Takahashi H, Maeda T, Umeda S, Suhara T, Mimura M, Kato M. It's not my fault: postdictive modulation of intentional binding by monetary gains and losses. PLoS One. 2012;7(12):e53421. doi: 10.1371/journal.pone.0053421. Epub 2012 Dec 28. | HCs |
| 36 | Wolpe N, Haggard P, Siebner HR, Rowe JB. Cue integration and the perception of action in intentional binding. Exp Brain Res. 2013;229(3):467-74. doi: 10.1007/s00221-013-3419-2. Epub 2013 Feb 1. | HCs |
| 37 | Moore JW, Teufel C, Subramaniam N, Davis G, Fletcher PC. Attribution of intentional causation influences the perception of observed movements: behavioral evidence and neural correlates. Front Psychol. 2013;4:23. doi: 10.3389/fpsyg.2013.00023. eCollection Jan 29 2013. | HCs |
| 38 | Parsons BD, Novich SD, Eagleman DM. Motor-sensory recalibration modulates perceived simultaneity of cross-modal events at different distances. Front Psychol. 2013;4:46. doi: 10.3389/fpsyg.2013.00046. eCollection Feb 26 2013. | HCs |
| 39 | Poonian SK, Cunnington R. Intentional binding in self-made and observed actions. Exp Brain Res. 2013;229(3):419-27. doi: 10.1007/s00221-013-3505-5. Epub 2013 Apr 11. | HCs |
| 40 | Faro D, McGill AL, Hastie R. The influence of perceived causation on judgments of time: an integrative review and implications for decision-making. Front Psychol. 2013;4:217. doi: 10.3389/fpsyg.2013.00217. eCollection May 14 2013. | Review |
| 41 | Vinding MC, Pedersen MN, Overgaard M. Unravelling intention: distal intentions increase the subjective sense of agency. Conscious Cogn. 2013 Sep;22(3):810-5. doi: 10.1016/j.concog.2013.05.003. Epub 2013 Jun 1. Epub 23732190 | HCs |
| 42 | Kawabe T, Roseboom W, Nishida S. The sense of agency is action-effect causality perception based on cross-modal grouping. Proc Biol Sci. 2013;280(1763):20130991. doi: 10.1098/rspb.2013.0991. Print 2013 Jul 22; Epub Jun 5 2013. | HCs |
| **43** | **Sperduti M, Pieron M, Leboyer M, Zalla T. Altered pre-reflective sense of agency in autism spectrum disorders as revealed by reduced intentional binding. J Autism Dev Disord. 2014;44(2):343-52. doi: 10.1007/s10803-013-1891-y.** | **Included** |
| 44 | Rohde M, Greiner L, Ernst MO. Asymmetries in visuomotor recalibration of time perception: does causal binding distort the window of integration? Acta Psychol (Amst). 2014;147:127-35. doi: 10.1016/j.actpsy.2013.07.011. Epub 2013 Aug 5. | HCs |
| 45 | Barlas Z, Obhi SS. Freedom, choice, and the sense of agency. Front Hum Neurosci. 2013;7:514. doi: 10.3389/fnhum.2013.00514. eCollection Aug 29 2013. | HCs |
| 46 | Cravo AM, Haddad H, Claessens PM, Baldo MV. Bias and learning in temporal binding: intervals between actions and outcomes are compressed by prior bias. Conscious Cogn. 2013;22(4):1174-80. doi: 10.1016/j.concog.2013.08.001. Epub 2013 Sep 7. | HCs |
| 47 | Frith CD. Action, agency and responsibility. Neuropsychologia. 2014;55:137-42. doi: 10.1016/j.neuropsychologia.2013.09.007. Epub 2013 Sep 11. | Opinion |
| 48 | Demanet J, Muhle-Karbe PS, Lynn MT, Blotenberg I, Brass M. Power to the will: how exerting physical effort boosts the sense of agency. Cognition. 2013;129(3):574-8. doi: 10.1016/j.cognition.2013.08.020. Epub 2013 Sep 21. | HCs |
| 49 | Yoshie M, Haggard P. Negative emotional outcomes attenuate sense of agency over voluntary actions. Curr Biol. 2013;23(20):2028-32. doi: 10.1016/j.cub.2013.08.034. Epub 2013 Oct 3. | HCs |
| 50 | Martin JR. Experiences of activity and causality in schizophrenia: when predictive deficits lead to a retrospective over-binding. Conscious Cogn. 2013;22(4):1361-74. doi: 10.1016/j.concog.2013.09.003. Epub 2013 Oct 3. | Review |
| **51** | **Wolpe N, Moore JW, Rae CL, Rittman T, Altena E, Haggard P, Rowe JB. The medial frontal-prefrontal network for altered awareness and control of action in corticobasal syndrome. Brain. 2014;137(Pt 1):208-20. doi: 10.1093/brain/awt302. Epub 2013 Nov 29.** | **Included** |
| 52 | Jo HG, Wittmann M, Hinterberger T, Schmidt S. The readiness potential reflects intentional binding. Front Hum Neurosci. 2014;8:421. doi: 10.3389/fnhum.2014.00421. eCollection Jun 10 2014. | HCs |
| 53 | Wolpe N, Rowe JB. Beyond the "urge to move": objective measures for the study of agency in the post-Libet era. Front Hum Neurosci. 2014;8:450. doi: 10.3389/fnhum.2014.00450. eCollection Jun 20 2014. | Review |
| 54 | Barlas Z, Obhi SS. Cultural background influences implicit but not explicit sense of agency for the production of musical tones. Conscious Cogn. 2014;28:94-103. doi: 10.1016/j.concog.2014.06.013. Epub 2014 Jul 20. | HCs |
| 55 | Cavazzana A, Begliomini C, Bisiacchi PS. Intentional binding effect in children: insights from a new paradigm. Front Hum Neurosci. 2014;8:651. doi: 10.3389/fnhum.2014.00651. eCollection Aug 25 2014. | HCs |
| 56 | Haering C, Kiesel A. Intentional Binding is independent of the validity of the action effect's identity. Acta Psychol (Amst). 2014;152:109-19. doi: 10.1016/j.actpsy.2014.07.015. Epub 2014 Sep 7. | HCs |
| 57 | Pfister R, Obhi SS, Rieger M, Wenke D. Action and perception in social contexts: intentional binding for social action effects. Front Hum Neurosci. 2014;8:667. doi: 10.3389/fnhum.2014.00667. eCollection Sep 2 2014. | HCs |
| 58 | Poonian SK, McFadyen J, Ogden J, Cunnington R. Implicit agency in observed actions: evidence for N1 suppression of tones caused by self-made and observed actions. J Cogn Neurosci. 2015;27(4):752-64. doi: 10.1162/jocn_a_00745. Epub 2014 Oct 16. | HCs |
| 59 | Braun N, Thorne JD, Hildebrandt H, Debener S. Interplay of agency and ownership: the intentional binding and rubber hand illusion paradigm combined. PLoS One. 2014;9(11):e111967. doi: 10.1371/journal.pone.0111967. eCollection Nov 4 2014. | HCs |
| 60 | Kumar N, Manjaly JA, Sunny MM. The relationship between action-effect monitoring and attention capture. J Exp Psychol Gen. 2015;144(1):18-23. doi: 10.1037/xge0000032. Epub 2014 Nov 10. | HCs |
| 61 | White PA. The pre-reflective experience of "I" as a continuously existing being: the role of temporal functional binding. Conscious Cogn. 2015;31:98-114. doi: 10.1016/j.concog.2014.11.003. Epub 2014 Nov 27. | Review |
| 62 | Lynn MT, Muhle-Karbe PS, Aarts H, Brass M. Priming determinist beliefs diminishes implicit (but not explicit) components of self-agency. Front Psychol. 2014;5:1483. doi: 10.3389/fpsyg.2014.01483. eCollection Dec 17 2014. | HCs |
| 63 | Caspar EA, Cleeremans A, Haggard P. The relationship between human agency and embodiment. Conscious Cogn. 2015;33:226-36. doi: 10.1016/j.concog.2015.01.007. Epub 2015 Feb 2. | HCs |
| 64 | Hascalovitz AC, Obhi SS. Personality and intentional binding: an exploratory study using the narcissistic personality inventory. Front Hum Neurosci. 2015;9:13. doi: 10.3389/fnhum.2015.00013. eCollection Feb 5 2015. | HCs |
| 65 | Yabe Y, Goodale MA. Time flies when we intend to act: temporal distortion in a go/no-go task. J Neurosci. 2015;35(12):5023-9. doi: 10.1523/JNEUROSCI.4386-14.2015. Epub Mar 25 2015. | HCs |
| 66 | Venkatasubramanian G. Understanding schizophrenia as a disorder of consciousness: biological correlates and translational implications from quantum theory perspectives. Clin Psychopharmacol Neurosci. 2015;13(1):36-47. doi: 10.9758/cpn.2015.13.1.36. Epub Apr 30 2015. | Review |
| 67 | Cavazzana A, Penolazzi B, Begliomini C, Bisiacchi PS. Neural underpinnings of the 'agent brain': new evidence from transcranial direct current stimulation. Eur J Neurosci. 2015;42(3):1889-94. doi: 10.1111/ejn.12937. Epub 2015 May 28. | HCs |
| 68 | Khalighinejad N, Haggard P. Modulating human sense of agency with non-invasive brain stimulation. Cortex. 2015;69:93-103. doi: 10.1016/j.cortex.2015.04.015. Epub 2015 May 2. | HCs |
| 69 | Dimaggio G, Lysaker PH. Commentary: "Personality and Intentional Binding: An Exploratory Study Using the Narcissistic Personality Inventory". Front Hum Neurosci. 2015;9:325. doi: 10.3389/fnhum.2015.00325. eCollection Jun 3 2015. | Opinion |
| 70 | Wen W, Yamashita A, Asama H. The influence of action-outcome delay and arousal on sense of agency and the intentional binding effect. Conscious Cogn. 2015;36:87-95. doi: 10.1016/j.concog.2015.06.004. Epub 2015 Jun 19. | HCs |
| 71 | Vinding MC, Jensen M, Overgaard M. The time between intention and action affects the experience of action. Front Hum Neurosci. 2015;9:366. doi: 10.3389/fnhum.2015.00366. eCollection Jun 19 2015. | HCs |
| 72 | Saito N, Takahata K, Murai T, Takahashi H. Discrepancy between explicit judgement of agency and implicit feeling of agency: Implications for sense of agency and its disorders. Conscious Cogn. 2015;37:1-7. doi: 10.1016/j.concog.2015.07.011. Epub 2015 Aug 5. | HCs |
| 73 | Zalla T, Sperduti M. The sense of agency in autism spectrum disorders: a dissociation between prospective and retrospective mechanisms? Front Psychol. 2015;6:1278. doi: 10.3389/fpsyg.2015.01278. eCollection Sep 8 2015. | Review |
| 74 | Kawabe T. Delayed visual feedback of one's own action promotes sense of control for auditory events. Front Integr Neurosci. 2015;9:57. doi: 10.3389/fnint.2015.00057. eCollection Nov 19 2015. | HCs |
| 75 | Capozzi F, Becchio C, Garbarini F, Savazzi S, Pia L. Temporal perception in joint action: This is MY action. Conscious Cogn. 2016;40:26-33. doi: 10.1016/j.concog.2015.12.004. Epub 2015 Dec 29. | HCs |
| 76 | Khalighinejad N, Di Costa S, Haggard P. Endogenous action selection processes in dorsolateral prefrontal cortex contribute to sense of agency: A meta-analysis of tDCS studies of 'intentional binding'. Brain Stimul. 2016;9(3):372-379. doi: 10.1016/j.brs.2016.01.005. Epub 2016 Jan 21. Epub 26896324 Free article. | Review |
| 77 | Zhao K, Hu L, Qu F, Cui Q, Piao Q, Xu H, Li Y, Wang L, Fu X. Voluntary action and tactile sensory feedback in the intentional binding effect. Exp Brain Res. 2016;234(8):2283-92. doi: 10.1007/s00221-016-4633-5. Epub 2016 Apr 1. | HCs |
| 78 | Christensen JF, Yoshie M, Di Costa S, Haggard P. Emotional valence, sense of agency and responsibility: A study using intentional binding. Conscious Cogn. 2016;43:1-10. doi: 10.1016/j.concog.2016.02.016. Epub 2016 May 9. | HCs |
| 79 | Tobias-Webb J, Limbrick-Oldfield EH, Gillan CM, Moore JW, Aitken MR, Clark L. Let me take the wheel: Illusory control and sense of agency. Q J Exp Psychol (Hove). 2017;70(8):1732-1746. doi: 10.1080/17470218.2016.1206128. Epub 2016 Jul 20. | HCs |
| 80 | Khalighinejad N, Haggard P. Extending experiences of voluntary action by association. Proc Natl Acad Sci U S A. 2016;113(31):8867-72. doi: 10.1073/pnas.1521223113. Epub 2016 Jul 19. | HCs |
| **81** | **Graham-Schmidt KT, Martin-Iverson MT, Holmes NP, Waters FAV. When one's sense of agency goes wrong: Absent modulation of time perception by voluntary actions and reduction of perceived length of intervals in passivity symptoms in schizophrenia. Conscious Cogn. 2016;45:9-23. doi: 10.1016/j.concog.2016.08.006. Epub 2016 Aug 18.** | **Included** |
| 82 | Howard EE, Edwards SG, Bayliss AP. Physical and mental effort disrupts the implicit sense of agency. Cognition. 2016;157:114-125. doi: 10.1016/j.cognition.2016.08.018. Epub 2016 Sep 6. | HCs |
| 83 | Khalighinejad N, Bahrami B, Caspar EA, Haggard P. Social transmission of experience of agency: An experimental study. Front Psychol. 2016;7:1315. doi: 10.3389/fpsyg.2016.01315. eCollection Aug 30 2016. | HCs |
| 84 | Yabe Y, Dave H, Goodale MA. Temporal distortion in the perception of actions and events. Cognition. 2017;158:1-9. doi: 10.1016/j.cognition.2016.10.009. Epub 2016 Oct 20. | HCs |
| 85 | Lush P, Parkinson J, Dienes Z. Lush Illusory temporal binding in meditators. Mindfulness (N Y). 2016;7(6):1416-1422. doi: 10.1007/s12671-016-0583-z. Epub 2016 Aug 2. | HCs |
| 86 | Kumar D, Srinivasan N. Multi-scale control influences sense of agency: Investigating intentional binding using event-control approach. Conscious Cogn. 2017;49:1-14. doi: 10.1016/j.concog.2016.12.014. Epub 2017 Jan 12. | HCs |
| 87 | Ruess M, Thomaschke R, Kiesel A. The time course of intentional binding. Atten Percept Psychophys. 2017;79(4):1123-1131. doi: 10.3758/s13414-017-1292-y. | HCs |
| 88 | Beck B, Di Costa S, Haggard P. Having control over the external world increases the implicit sense of agency. Cognition. 2017;162:54-60. doi: 10.1016/j.cognition.2017.02.002. Epub 2017 Feb 14. | HCs |
| 89 | Khalighinejad N, Kunnumpurath A, Bertini C, Ladavas E, Haggard P. Subliminal modulation of voluntary action experience: A neuropsychological investigation. Cortex. 2017;90:58-70. doi: 10.1016/j.cortex.2017.02.012. Epub 2017 Mar 10. | HCs |
| 90 | Vastano R, Pozzo T, Brass M. The action congruency effect on the feelings of agency. Conscious Cogn. 2017;51:212-222. doi: 10.1016/j.concog.2017.04.002. Epub 2017 Apr 11. | HCs |
| 91 | Vuorre M, Metcalfe J. Voluntary action alters the perception of visual illusions. Atten Percept Psychophys. 2017;79(5):1495-1505. doi: 10.3758/s13414-017-1321-x. | HCs |
| **92** | **Ricciardi L, Haggard P, de Boer L, Sorbera C, Stenner MP, Morgante F, Edwards MJ. Acting without being in control: Exploring volition in Parkinson's disease with impulsive compulsive behaviours. Parkinsonism Relat Disord. 2017;40:51-57. doi: 10.1016/j.parkreldis.2017.04.011. Epub 2017 Apr 20.** | **Included** |
| 93 | Lush P, Caspar EA, Cleeremans A, Haggard P, Magalhães De Saldanha da Gama PA, Dienes Z. The power of suggestion: Posthypnotically induced changes in the temporal binding of intentional action outcomes. Psychol Sci. 2017;28(5):661-669. doi: 10.1177/0956797616687015. Epub 2017 Mar 16. | HCs |
| 94 | Cavazzana A, Begliomini C, Bisiacchi PS. Intentional binding as a marker of agency across the lifespan. Conscious Cogn. 2017;52:104-114. doi: 10.1016/j.concog.2017.04.016. Epub 2017 May 11. | HCs |
| 95 | Wen W, Yamashita A, Asama H. The influence of performance on action-effect integration in sense of agency. Conscious Cogn. 2017;53:89-98. doi: 10.1016/j.concog.2017.06.008. Epub 2017 Jun 23. | HCs |
| 96 | Ruess M, Thomaschke R, Haering C, Wenke D, Kiesel A. Intentional binding of two effects. Psychol Res. 2018;82(6):1102-1112. doi: 10.1007/s00426-017-0892-4. Epub 2017 Jul 8. | HCs |
| 97 | Di Costa S, Théro H, Chambon V, Haggard P. Try and try again: Post-error boost of an implicit measure of agency. Q J Exp Psychol (Hove). 2018;71(7):1584-1595. doi: 10.1080/17470218.2017.1350871. Epub 2018 Jan 1. | HCs |
| 98 | Goldberg M, Busch N, van der Meer E. The amount of recent action-outcome coupling modulates the mechanisms of the intentional binding effect: A behavioral and ERP study. Conscious Cogn. 2017;56:135-149. doi: 10.1016/j.concog.2017.07.001. Epub 2017 Jul 14. | HCs |
| 99 | Makwana M, Srinivasan N. Intended outcome expands in time. Sci Rep. 2017;7(1):6305. doi: 10.1038/s41598-017-05803-1. Epub Jul 24 2017. | HCs |
| 100 | Straube B, Schülke R, Drewing K, Kircher T, van Kemenade BM. Hemispheric differences in the processing of visual consequences of active vs. passive movements: a transcranial direct current stimulation study. Exp Brain Res. 2017;235(10):3207-3216. doi: 10.1007/s00221-017-5053-x. Epub 2017 Jul 31. | HCs |
| **101** | **Saito N, Takahata K, Yamakado H, Sawamoto N, Saito S, Takahashi R, Murai T, Takahashi H. Altered awareness of action in Parkinson's disease: evaluations by explicit and implicit measures. Sci Rep. 2017;7(1):8019. doi: 10.1038/s41598-017-08482-0. Epub Aug 14 2017.** | **Included** |
| 102 | Yoshie M, Haggard P. Effects of emotional valence on sense of agency require a predictive model. Sci Rep. 2017;7(1):8733. doi: 10.1038/s41598-017-08803-3. Epub Aug 18 2017. | HCs |
| 103 | Barlas Z, Hockley WE, Obhi SS. The effects of freedom of choice in action selection on perceived mental effort and the sense of agency. Acta Psychol (Amst). 2017;180:122-129. doi: 10.1016/j.actpsy.2017.09.004. Epub 2017 Sep 20. | HCs |
| 104 | Wang Y, Damen TGE, Aarts H. Uncovering effects of self-control and stimulus-driven action selection on the sense of agency. Conscious Cogn. 2017;55:245-253. doi: 10.1016/j.concog.2017.09.005. Epub 2017 Sep 22. | HCs |
| 105 | Matute H, Cubillas CP, Garaizar P. Learning to infer the time of our actions and decisions from their consequences. Conscious Cogn. 2017;56:37-49. doi: 10.1016/j.concog.2017.09.009. Epub 29045917 Free article. | HCs |
| 106 | Barlas Z, Hockley WE, Obhi SS. Effects of free choice and outcome valence on the sense of agency: evidence from measures of intentional binding and feelings of control. Exp Brain Res. 2018;236(1):129-139. doi: 10.1007/s00221-017-5112-3. Epub 2017 Oct 27. | HCs |
| 107 | Zopf R, Polito V, Moore J. Revisiting the link between body and agency: visual movement congruency enhances intentional binding but is not body-specific. Sci Rep. 2018;8(1):196. doi: 10.1038/s41598-017-18492-7. Epub Jan 9 2018. | HCs |
| 108 | Ruess M, Thomaschke R, Kiesel A. Intentional binding of visual effects. Atten Percept Psychophys. 2018;80(3):713-722. doi: 10.3758/s13414-017-1479-2. | HCs |
| 109 | Pyasik M, Burin D, Pia L. On the relation between body ownership and sense of agency: A link at the level of sensory-related signals. Acta Psychol (Amst). 2018;185:219-228. doi: 10.1016/j.actpsy.2018.03.001. Epub 2018 Mar 16. | HCs |
| 110 | Hughes G. The role of the temporoparietal junction in implicit and explicit sense of agency. Neuropsychologia. 2018;113:1-5. doi: 10.1016/j.neuropsychologia.2018.03.020. Epub 2018 Mar 19. | HCs |
| 111 | Barlas Z, Kopp S. Action choice and outcome congruency independently affect intentional binding and feeling of control judgments. Front Hum Neurosci. 2018;12:137. doi: 10.3389/fnhum.2018.00137. eCollection Apr 11 2018. | HCs |
| 112 | Thanopoulos V, Psarou E, Vatakis A. Robust intentional binding for causally-linked sequences of naturalistic events but not for abstract event sequences. Acta Psychol (Amst). 2018;190:159-173. doi: 10.1016/j.actpsy.2018.08.001. Epub 2018 Aug 15. | HCs |
| 113 | Caspar EA, Cleeremans A, Haggard P. Only giving orders? An experimental study of the sense of agency when giving or receiving commands. PLoS One. 2018;13(9):e0204027. doi: 10.1371/journal.pone.0204027. eCollection Sep 26 2018. | HCs |
| 114 | Majchrowicz B, Wierzchoń M. Unexpected action outcomes produce enhanced temporal binding but diminished judgement of agency. Conscious Cogn. 2018;65:310-324. doi: 10.1016/j.concog.2018.09.007. Epub 2018 Oct 11. | HCs |
| 115 | Imaizumi S, Tanno Y. Intentional binding coincides with explicit sense of agency. Conscious Cogn. 2019;67:1-15. doi: 10.1016/j.concog.2018.11.005. Epub 2018 Nov 21. | HCs |
| 116 | Kirsch W, Kunde W, Herbort O. Intentional binding is unrelated to action intention. J Exp Psychol Hum Percept Perform. 2019;45(3):378-385. doi: 10.1037/xhp0000612. Epub 2018 Dec 20. | HCs |
| 117 | Morioka S, Hayashida K, Nishi Y, Negi S, Nishi Y, Osumi M, Nobusako S. Changes in intentional binding effect during a novel perceptual-motor task. PeerJ. 2018;6:e6066. doi: 10.7717/peerj.6066. eCollection Dec 11 2018. | HCs |
| 118 | Schofield JS, Shell CE, Thumser ZC, Beckler DT, Nataraj R, Marasco PD. Characterization of the sense of agency over the actions of neural-machine interface-operated prostheses. J Vis Exp. 2019;(143). doi: 10.3791/58702. Epub Jan 7 2019. | HCs |
| 119 | Baizabal-Carvallo JF, Hallett M, Jankovic J. Pathogenesis and pathophysiology of functional (psychogenic) movement disorders. Neurobiol Dis. 2019;127:32-44. doi: 10.1016/j.nbd.2019.02.013. Epub 2019 Feb 21. | Review |
| 120 | Christensen JF, Di Costa S, Beck B, Haggard P. I just lost it! Fear and anger reduce the sense of agency: a study using intentional binding. Exp Brain Res. 2019;237(5):1205-1212. doi: 10.1007/s00221-018-5461-6. Epub 2019 Mar 2. | HCs |
| 121 | Ma K, Hommel B, Chen H. Context-induced contrast and assimilation effects in explicit and implicit measures of agency. Sci Rep. 2019;9(1):3883. doi: 10.1038/s41598-019-40545-2. Epub Mar 7 2019. | HCs |
| 122 | Render A, Jansen P. Dopamine and sense of agency: Determinants in personality and substance use. PLoS One. 2019;14(3):e0214069. doi: 10.1371/journal.pone.0214069. eCollection Mar 19 2019. | HCs |
| 123 | Lush P, Dienes Z. Time perception and the experience of agency in meditation and hypnosis. Psych J. 2019;8(1):36-50. doi: 10.1002/pchj.276. | Review |
| 124 | Malik RA, Obhi SS. Social exclusion reduces the sense of agency: Evidence from intentional binding. Conscious Cogn. 2019;71:30-38. doi: 10.1016/j.concog.2019.03.004. Epub 2019 Mar 27. | HCs |
| 125 | Suzuki K, Lush P, Seth AK, Roseboom W. Intentional binding without intentional action. Psychol Sci. 2019;30(6):842-853. doi: 10.1177/0956797619842191. Epub 2019 Apr 25. | HCs |
| 126 | Li M, Li LMW, Zhao K, Gao DG. Cultural group perception enhances sense of agency in a multicultural society. Scand J Psychol. 2019;60(4):394-403. doi: 10.1111/sjop.12552. Epub 2019 May 23. | HCs |
| 127 | Wen W. Does delay in feedback diminish sense of agency? A review. Conscious Cogn. 2019;73:102759. doi: 10.1016/j.concog.2019.05.007. Epub 2019 Jun 4. | Review |
| 128 | Lush P, Roseboom W, Cleeremans A, Scott RB, Seth AK, Dienes Z. Intentional binding as Bayesian cue combination: Testing predictions with trait individual differences. J Exp Psychol Hum Percept Perform. 2019;45(9):1206-1217. doi: 10.1037/xhp0000661. Epub 2019 Jun 20. | HCs |
| 129 | De Pirro S, Lush P, Parkinson J, Duka T, Critchley HD, Badiani A. Effect of alcohol on the sense of agency in healthy humans. Addict Biol. 2020;25(4):e12796. doi: 10.1111/adb.12796. Epub 2019 Jun 20. | HCs |
| 130 | Antusch S, Aarts H, Custers R. The role of intentional strength in shaping the sense of agency. Front Psychol. 2019;10:1124. doi: 10.3389/fpsyg.2019.01124. eCollection May 21 2019. | HCs |
| 131 | Imaizumi S, Tanno Y, Imamizu H. Compress global, dilate local: Intentional binding in action-outcome alternations. Conscious Cogn. 2019;73:102768. doi: 10.1016/j.concog.2019.102768. Epub 2019 Jun 26. | HCs |
| 132 | Di Plinio S, Arnò S, Perrucci MG, Ebisch SJH. Environmental control and psychosis-relevant traits modulate the prospective sense of agency in non-clinical individuals. Conscious Cogn. 2019;73:102776. doi: 10.1016/j.concog.2019.102776. Epub 2019 Jul 1. Epub 31272013 | HCs |
| 133 | Ulloa JL, Vastano R, George N, Brass M. The impact of eye contact on the sense of agency. Conscious Cogn. 2019;74:102794. doi: 10.1016/j.concog.2019.102794. Epub 2019 Jul 31. | HCs |
| 134 | Makwana M, Srinivasan N. Self-associated stimuli produce stronger intentional binding. J Exp Psychol Hum Percept Perform. 2019;45(11):1436-1442. doi: 10.1037/xhp0000687. Epub 2019 Aug 22. | HCs |
| 135 | Legaspi R, Toyoizumi T. A Bayesian psychophysics model of sense of agency. Nat Commun. 2019;10(1):4250. doi: 10.1038/s41467-019-12170-0. Epub Sep 18 2019. | HCs |
| 136 | Barlas Z. When robots tell you what to do: Sense of agency in human- and robot-guided actions. Conscious Cogn. 2019;75:102819. doi: 10.1016/j.concog.2019.102819. Epub 2019 Sep 18. | HCs |
| 137 | Grynszpan O, Sahaï A, Hamidi N, Pacherie E, Berberian B, Roche L, Saint-Bauzel L. The sense of agency in human-human vs human-robot joint action. Conscious Cogn. 2019;75:102820. doi: 10.1016/j.concog.2019.102820. Epub 2019 Sep 24. | HCs |
| 138 | Ruess M, Thomaschke R, Kiesel A. Acting and reacting: Is intentional binding due to sense of agency or to temporal expectancy? J Exp Psychol Hum Percept Perform. 2020;46(1):1-9. doi: 10.1037/xhp0000700. Epub 2019 Nov 7. | HCs |
| 139 | Antusch S, Custers R, Marien H, Aarts H. Intentionality and temporal binding: Do causality beliefs increase the perceived temporal attraction between events? Conscious Cogn. 2020;77:102835. doi: 10.1016/j.concog.2019.102835. Epub 2019 Nov 6. | HCs |
| 140 | Faivre N, Vuillaume L, Bernasconi F, Salomon R, Blanke O, Cleeremans A. Sensorimotor conflicts alter metacognitive and action monitoring. Cortex. 2020;124:224-234. doi: 10.1016/j.cortex.2019.12.001. Epub 2019 Dec 19. | HCs |
| 141 | Vastano R, Ambrosini E, Ulloa JL, Brass M. Action selection conflict and intentional binding: An ERP study. Cortex. 2020;126:182-199. doi: 10.1016/j.cortex.2020.01.013. Epub 2020 Jan 31. | HCs |
| 142 | Di Plinio S, Arnò S, Perrucci MG, Ebisch SJH. The evolving sense of agency: Context recency and quality modulate the interaction between prospective and retrospective processes. Conscious Cogn. 2020;80:102903. doi: 10.1016/j.concog.2020.102903. Epub 2020 Mar 4. | HCs |
| 143 | Lorimer S, McCormack T, Blakey E, Lagnado DA, Hoerl C, Tecwyn EC, Buehner MJ. The developmental profile of temporal binding: From childhood to adulthood. Q J Exp Psychol (Hove). 2020;73(10):1575-1586. doi: 10.1177/1747021820925075. Epub 2020 Jun 2. | HCs |
| 144 | Nataraj R, Sanford S, Shah A, Liu M. Agency and performance of reach-to-grasp with modified control of a virtual hand: Implications for rehabilitation. Front Hum Neurosci. 2020;14:126. doi: 10.3389/fnhum.2020.00126. eCollection Apr 23 2020. | HCs |
| 145 | Hoerl C, Lorimer S, McCormack T, Lagnado DA, Blakey E, Tecwyn EC, Buehner MJ. Temporal binding, causation, and agency: developing a new theoretical framework. Cogn Sci. 2020;44(5):e12843. doi: 10.1111/cogs.12843. | HCs |
| 146 | Nataraj R, Hollinger D, Liu M, Shah A. Disproportionate positive feedback facilitates sense of agency and performance for a reaching movement task with a virtual hand. PLoS One. 2020;15(5):e0233175. doi: 10.1371/journal.pone.0233175. eCollection May 20 2020. | HCs |
| 147 | Endo S, Fröhner J, Musić S, Hirche S, Beckerle P. Effect of external force on agency in physical human-machine interaction. Front Hum Neurosci. 2020;14:114. doi: 10.3389/fnhum.2020.00114. eCollection May 8 2020. | HCs |
| 148 | Cubillas CP, Landáburu Í, Matute H. Methodological factors involved in the study of temporal binding using the open source software Labclock Web. Front Psychol. 2020;11:1040. doi: 10.3389/fpsyg.2020.01040. eCollection May 27 2020. | HCs |
| 149 | Di Plinio S, Perrucci MG, Ebisch SJH. The prospective sense of agency is rooted in local and global properties of intrinsic functional brain networks. J Cogn Neurosci. 2020;32(9):1764-1779. doi: 10.1162/jocn_a_01590. Epub 2020 Jun 12. | HCs |
| **150** | **Möller TJ, Braun N, Thöne AK, Herrmann CS, Philipsen A. The senses of agency and ownership in patients with borderline personality disorder. Front Psychiatry. 2020;11:474. doi: 10.3389/fpsyt.2020.00474. eCollection Jun 9 2020.** | **Included** |
| 151 | Soral W, Kofta M, Bukowski M. Helplessness experience and intentional (un-)binding: Control deprivation disrupts the implicit sense of agency. J Exp Psychol Gen. 2021;150(2):289-305. doi: 10.1037/xge0000791. Epub 2020 Jul 13. | HCs |
| 152 | Pansardi O, Pyasik M, Pia L. Musical expertise affects the sense of agency: Intentional binding in expert pianists. Conscious Cogn. 2020;84:102984. doi: 10.1016/j.concog.2020.102984. Epub 2020 Jul 14. | HCs |
| 153 | Lafleur A, Soulières I, Forgeot d'Arc B. Sense of agency: Sensorimotor signals and social context are differentially weighed at implicit and explicit levels. Conscious Cogn. 2020;84:103004. doi: 10.1016/j.concog.2020.103004. Epub 2020 Aug 17. | HCs |
| 154 | Yamamoto K. Cue integration as a common mechanism for action and outcome bindings. Cognition. 2020;205:104423. doi: 10.1016/j.cognition.2020.104423. Epub 2020 Aug 22. | HCs |
| 155 | Herman AM, Tsakiris M. Feeling in control: The role of cardiac timing in the sense of agency. Affect Sci. 2020;1(3):155-171. doi: 10.1007/s42761-020-00013-x. | HCs |
| 156 | Hayashida K, Nishi Y, Masuike A, Morioka S. Intentional binding effects in the experience of noticing the regularity of a perceptual-motor task. Brain Sci. 2020;10(9):659. doi: 10.3390/brainsci10090659. Epub Sep 22 2020. | HCs |
| 157 | Majchrowicz B, Kulakova E, Di Costa S, Haggard P. Learning from informative losses boosts the sense of agency. Q J Exp Psychol (Hove). 2020;73(12):2272-2289. doi: 10.1177/1747021820958258. Epub 2020 Sep 24. | HCs |
| 158 | Render A, Jansen P. Influence of arousal on intentional binding: Impaired action binding, intact outcome binding. Atten Percept Psychophys. 2021;83(1):103-113. doi: 10.3758/s13414-020-02105-z. | HCs |
| 159 | Melcher D, Kumar D, Srinivasan N. The role of action intentionality and effector in the subjective expansion of temporal duration after saccadic eye movements. Sci Rep. 2020;10(1):16922. doi: 10.1038/s41598-020-73830-6. Epub Oct 9 2020. | HCs |
| 160 | Muth FV, Wirth R, Kunde W. Temporal binding past the Libet clock: testing design factors for an auditory timer. Behav Res Methods. 2021;53(3):1322-1341. doi: 10.3758/s13428-020-01474-5. | HCs |
| 161 | Seghezzi S, Zapparoli L. Predicting the sensory consequences of self-generated actions: pre-supplementary motor area as supra-modal hub in the sense of agency experience. Brain Sci. 2020;10(11):825. doi: 10.3390/brainsci10110825. Epub Nov 7 2020. | HCs |
| 162 | Aytemur A, Levita L. A reduction in the implicit sense of agency during adolescence compared to childhood and adulthood. Conscious Cogn. 2021;87:103060. doi: 10.1016/j.concog.2020.103060. Epub 2020 Dec 1. | HCs |
| 163 | Jenkins M, Esemezie O, Lee V, Mensingh M, Nagales K, Obhi SS. An investigation of "We" agency in co-operative joint actions. Psychol Res. 2021. doi: 10.1007/s00426-020-01462-6. Online ahead of print Jan 4 2021. | HCs |
| **164** | **Zapparoli L, Seghezzi S, Devoto F, Mariano M, Banfi G, Porta M, Paulesu E. Altered sense of agency in Gilles de la Tourette syndrome: behavioural, clinical and functional magnetic resonance imaging findings. Brain Commun. 2020;2(2):fcaa204. doi: 10.1093/braincomms/fcaa204. eCollection Nov 19 2020.** | **Included** |
| 165 | Antusch S, Custers R, Marien H, Aarts H. Intentional action and limitation of personal autonomy. Do restrictions of action selection decrease the sense of agency? Conscious Cogn. 2021;88:103076. doi: 10.1016/j.concog.2021.103076. Epub 2021 Jan 20. | HCs |
| 166 | Nataraj R, Sanford S. Control modification of grasp force covaries agency and performance on rigid and compliant surfaces. Front Bioeng Biotechnol. 2021;8:574006. doi: 10.3389/fbioe.2020.574006. eCollection Jan 13 2020. | HCs |
| 167 | Hayashida K, Nishi Y, Osumi M, Nobusako S, Morioka S. Goal sharing with others modulates the sense of agency and motor accuracy in social contexts. PLoS One. 2021;16(2):e0246561. doi: 10.1371/journal.pone.0246561. eCollection Feb 4 2021. | HCs |
| 168 | Schmidt S, Wagner G, Walter M, Stenner MP. A psychophysical window onto the subjective experience of compulsion. Brain Sci. 2021;11(2):182. doi: 10.3390/brainsci11020182. Epub Feb 2 2021. | Review |
| 169 | Liu M, Wilder S, Sanford S, Saleh S, Harel NY, Nataraj R. Training with agency-inspired feedback from an instrumented glove to improve functional grasp performance. Sensors (Basel). 2021;21(4):1173. doi: 10.3390/s21041173. Epub Feb 7 2021. | HCs |
| 170 | Rashidi M, Schmitgen MM, Weisbrod M, Schnell K, Wolf RC, Whittington MA. Right parietotemporal activity predicts sense of agency under uncertain delays of sensory outcomes. J Neurophysiol. 2021;125(3):903-914. doi: 10.1152/jn.00640.2020. Epub 2021 Feb 10. | HCs |
| **171** | **Finnemann JJS, Plaisted-Grant K, Moore J, Teufel C, Fletcher PC. Low-level, prediction-based sensory and motor processes are unimpaired in Autism. Neuropsychologia. 2021;156:107835. doi: 10.1016/j.neuropsychologia.2021.107835. Epub 2021 Mar 29.** | **Included** |
| 172 | Antusch S, Custers R, Marien H, Aarts H. Studying the sense of agency in the absence of motor movement: an investigation into temporal binding of tactile sensations and auditory effects. Exp Brain Res. 2021. doi: 10.1007/s00221-021-06087-8. Online ahead of print Apr 7 2021. | HCs |
| 173 | Dubynin IA, Yashin AS, Velichkovsky BM, Shishkin SL. An experimental paradigm for studying sense of agency in joint human-machine motor actions. Exp Brain Res. 2021. doi: 10.1007/s00221-021-06105-9. Online ahead of print Apr 20 2021. | HCs |
| 174 | Sarma D, Srinivasan N. Intended emotions influence intentional binding with emotional faces: Larger binding for intended negative emotions. Conscious Cogn. 2021;92:103136. doi: 10.1016/j.concog.2021.103136. Epub 2021 May 6. | HCs |
| 175 | Ma K, Qu J, Yang L, Zhao W, Hommel B. Explicit and implicit measures of body ownership and agency: affected by the same manipulations and yet independent. Exp Brain Res. 2021. doi: 10.1007/s00221-021-06125-5. Online ahead of print May 11 2021. | HCs |
| 176 | Qu J, Ma K, Hommel B. Cognitive load dissociates explicit and implicit measures of body ownership and agency. Psychon Bull Rev. 2021. doi: 10.3758/s13423-021-01931-y. Online ahead of print May 25 2021. | HCs |
| 177 | Aytemur A, Lee KH, Levita L. Neural correlates of implicit agency during the transition from adolescence to adulthood: An ERP study. Neuropsychologia. 2021;158:107908. doi: 10.1016/j.neuropsychologia.2021.107908. Online ahead of print May 29 2021. | HCs |
| 178 | Jenkins M, Obhi SS. Exploring the relationship between perceived Action-Outcome distance and Agency: Evidence from temporal binding. Conscious Cogn. 2021;94:103177. doi: 10.1016/j.concog.2021.103177. Epub 2021 Jul 29. | HCs |
| 179 | Roselli C, Ciardo F, Wykowska A. Intentions with actions: The role of intentionality attribution on the vicarious sense of agency in Human-Robot interaction. Q J Exp Psychol (Hove). 2022;75(4):616-632. doi: 10.1177/17470218211042003. Epub 2021 Sep 2. | HCs |
| 180 | Galang CM, Malik R, Kinley I, Obhi SS. Studying sense of agency online: Can intentional binding be observed in uncontrolled online settings? Conscious Cogn. 2021;95:103217. doi: 10.1016/j.concog.2021.103217. Epub 2021 Oct 5. | HCs |
| 181 | Lopez-Sola E, Moreno-Bote R, Arsiwalla XD. Sense of agency for mental actions: Insights from a belief-based action-effect paradigm. Conscious Cogn. 2021;96:103225. doi: 10.1016/j.concog.2021.103225. Epub 2021 Oct 21. | HCs |
| 182 | Qu J, Sun Y, Yang L, Hommel B, Ma K. Physical load reduces synchrony effects on agency and ownership in the virtual hand illusion. Conscious Cogn. 2021;96:103227. doi: 10.1016/j.concog.2021.103227. Epub 2021 Oct 28. | HCs |
| 183 | Reddy NN. The implicit sense of agency is not a perceptual effect but is a judgment effect. Cogn Process. 2022;23(1):1-13. doi: 10.1007/s10339-021-01066-x. Epub 2021 Nov 9. | Review |
| 184 | Brandt F, Ullrich M, Laube M, Kopka K, Bachmann M, Löser R, Pietzsch J, Pietzsch HJ, van den Hoff J, Wodtke R. "Clickable" albumin binders for modulating the tumor uptake of targeted radiopharmaceuticals. J Med Chem. 2022;65(1):710-733. doi: 10.1021/acs.jmedchem.1c01791. Epub 2021 Dec 23. | Unrelated |
| 185 | Nataraj R, Sanford S, Liu M, Harel NY. Hand dominance in the performance and perceptions of virtual reach control. Acta Psychol (Amst). 2022;223:103494. doi: 10.1016/j.actpsy.2022.103494. Epub 2022 Jan 16. | HCs |
| 186 | Siebertz M, Jansen P. Diverging implicit measurement of sense of agency using interval estimation and Libet clock. Conscious Cogn. 2022;99:103287. doi: 10.1016/j.concog.2022.103287. Epub 2022 Feb 4. | HCs |
| 187 | Muth FV, Wirth R, Kunde W. Temporal binding in multi-step action-event sequences is driven by altered effect perception. Conscious Cogn. 2022;99:103299. doi: 10.1016/j.concog.2022.103299. Epub 2022 Feb 17. | HCs |
| 188 | Reis M, Weller L, Muth FV. To follow or not to follow: Influence of valence and consensus on the sense of agency. Conscious Cogn. 2022;102:103347. doi: 10.1016/j.concog.2022.103347. Epub 2022 May 13. | HCs |
| 189 | Niikuni K, Nakanishi M, Sugiura M. Intentional binding and self-transcendence: Searching for pro-survival behavior in sense-of-agency. Conscious Cogn. 2022;102:103351. doi: 10.1016/j.concog.2022.103351. Epub 2022 May 20. | HCs |
| **190** | **Engel MM, Ainley V, Tsakiris M, Chris Dijkerman H, Keizer A. Sense of agency during and following recovery from anorexia nervosa. Conscious Cogn. 2022;103:103369. doi: 10.1016/j.concog.2022.103369. Epub 2022 Jun 16.** | **Included** |
| 191 | Malik RA, Galang CM, Finger E. The sense of agency for brain disorders: A comprehensive review and proposed framework. Neurosci Biobehav Rev. 2022;139:104759. doi: 10.1016/j.neubiorev.2022.104759. Epub 2022 Jun 30. | Review |
| 192 | Ohata R, Asai T, Imaizumi S, Imamizu H. I hear my voice; Therefore I spoke: The sense of agency over speech is enhanced by hearing one's own voice. Psychol Sci. 2022;33(8):1226-1239. doi: 10.1177/09567976211068880. Epub 2022 Jul 5. | HCs |
| 193 | Jenkins M, Obhi SS. Mistakes strengthen the temporal binding effect in the context of goal-directed actions. Exp Brain Res. 2022;240(7-8):2191-2203. doi: 10.1007/s00221-022-06407-6. Epub 2022 Jul 7. | HCs |
| 194 | Koreki A, Goeta D, Ricciardi L, Eilon T, Chen J, Critchley HD, Garfinkel SN, Edwards M, Yogarajah M. The relationship between interoception and agency and its modulation by heartbeats: an exploratory study. Sci Rep. 2022;12(1):13624. doi: 10.1038/s41598-022-16569-6. Epub 2022 Aug 10. | HCs |
| 195 | Roselli C, Ciardo F, De Tommaso D, Wykowska A. Human-likeness and attribution of intentionality predict vicarious sense of agency over humanoid robot actions. Sci Rep. 2022;12(1):13845. doi: 10.1038/s41598-022-18151-6. Epub 2022 Aug 16. | HCs |
| 196 | Kirsch W, Kunde W. On the role of interoception in body and object perception: A multisensory-integration account. Perspect Psychol Sci. 2022:17456916221096138. doi: 10.1177/17456916221096138. Epub ahead of print 2022 Aug 22. | Review |
| 197 | Sugiura M. Adaptability, supernaturalness, and the neurocognitive basis of the self-transcendence trait: Toward an integrated framework through disaster psychology and a self-agency model. Front Behav Neurosci. 2022;16:943809. doi: 10.3389/fnbeh.2022.943809. Epub 2022 Aug 18. | Review |
| 198 | Scott NJ, Ghanem M, Beck B, Martin AK. Depressive traits are associated with a reduced effect of choice on intentional binding. Conscious Cogn. 2022;105:103412. doi: 10.1016/j.concog.2022.103412. Epub 2022 Sep 7. | HCs |
| 199 | University College London. The Pathophysiology of Functional Neurological Disorders. ClinicalTrials.gov Identifier: NCT02905877; September 2016-September 30, 2019. | No data |
| 200 | Istituto Ortopedico Galeazzi (Eraldo Paulescu). The Sense of Agency (SOA). ClinicalTrials.gov Identifier: NCT03361332; April 2017-December 19, 2019. | No data |
| 201 | Stetson C, Cui X, Montague PR, Eagleman DM. Motor-sensory recalibration leads to an illusory reversal of action and sensation. Neuron. 2006;51(5):651-9. doi: 10.1016/j.neuron.2006.08.006. | HCs |
| **202** | **Haggard P, Martin F, Taylor-Clarke M, Jeannerod M, Franck N. Awareness of action in schizophrenia. Neuroreport. 2003;14(7):1081-5. doi: 10.1097/01.wnr.0000073684. 00308.c0.** | **Included** |
| **203** | **Voss M, Moore J, Hauser M, Gallinat J, Heinz A, Haggard P. Altered awareness of action in schizophrenia: a specific deficit in predicting action consequences. Brain. 2010 ;133(10):3104-12. doi: 10.1093/brain/awq152. Epub 2010 Aug 4.** | **Included** |
| **204** | **Kranick SM, Moore JW, Yusuf N, Martinez VT, LaFaver K, Edwards MJ, Mehta AR, Collins P, Harrison NA, Haggard P, Hallett M, Voon V. Action-effect binding is decreased in motor conversion disorder: implications for sense of agency. Mov Disord. 2013;28(8):1110-6. doi: 10.1002/mds.25408. Epub 2013 Mar 14.** | **Included** |
| **205** | **Franck N, Posada A, Pichon S, Haggard P. Altered subjective time of events in schizophrenia. J Nerv Ment Dis. 2005;193(5):350-3. doi: 10.1097/01.nmd.0000161699.76032.09.** | **Included** |
| **206** | **Moore JW, Schneider SA, Schwingenschuh P, Moretto G, Bhatia KP, Haggard P. Dopaminergic medication boosts action-effect binding in Parkinson's disease. Neuropsychologia. 2010;48(4):1125-32. doi: 10.1016/j.neuropsychologia.2009.12.014. Epub 2009 Dec 16.** | **Included** |
| 207 | Hauser M, Knoblich G, Repp BH, Lautenschlager M, Gallinat J, Heinz A, Voss M. Altered sense of agency in schizophrenia and the putative psychotic prodrome. Psychiatry Res. 2011a;186(2-3):170-6. doi: 10.1016/j.psychres.2010.08.003. Epub 2010 Sep 9. | No IB |
| **208** | **Hauser M, Moore JW, de Millas W, Gallinat J, Heinz A, Haggard P, Voss M. Sense of agency is altered in patients with a putative psychotic prodrome. Schizophr Res. 2011b;126(1-3):20-7. doi: 10.1016/j.schres.2010.10.031. Epub 2010 Nov 26.** | **Included** |

**Table S2**. Risk of bias for included studies according to the Agency for Healthcare Research and Quality (2014) criteria.

|  | **Study** | **Risk of bias** | **Level** |
| --- | --- | --- | --- |
|  | *Haggard et al., 2003* | Selection | Low |
|  |  |  | Medium |
|  |  |  | High |
|  |  | Performance | Low |
|  |  |  | Medium |
|  |  |  | High |
|  |  | Detection | Low |
|  |  |  | Medium |
|  |  |  | High |
|  |  | Attrition | Low |
|  |  |  | Medium |
|  |  |  | High |
|  |  | Reporting | Low |
|  |  |  | Medium |
|  |  |  | High |
|  | *Franck et al., 2005* | Selection | Low |
|  |  |  | Medium |
|  |  |  | High |
|  |  | Performance | Low |
|  |  |  | Medium |
|  |  |  | High |
|  |  | Detection | Low |
|  |  |  | Medium |
|  |  |  | High |
|  |  | Attrition | Low |
|  |  |  | Medium |
|  |  |  | High |
|  |  | Reporting | Low |
|  |  |  | Medium |
|  |  |  | High |
|  | *Moore et al., 2010* | Selection | Low |
|  |  |  | Medium |
|  |  |  | High |
|  |  | Performance | Low |
|  |  |  | Medium |
|  |  |  | High |
|  |  | Detection | Low |
|  |  |  | Medium |
|  |  |  | High |
|  |  | Attrition | Low |
|  |  |  | Medium |
|  |  |  | High |
|  |  | Reporting | Low |
|  |  |  | Medium |
|  |  |  | High |
|  | *Voss et al., 2010* | Selection | Low |
|  |  |  | Medium |
|  |  |  | High |
|  |  | Performance | Low |
|  |  |  | Medium |
|  |  |  | High |
|  |  | Detection | Low |
|  |  |  | Medium |
|  |  |  | High |
|  |  | Attrition | Low |
|  |  |  | Medium |
|  |  |  | High |
|  |  | Reporting | Low |
|  |  |  | Medium |
|  |  |  | High |
|  | *Hauser et al., 2011* | Selection | Low |
|  |  |  | Medium |
|  |  |  | High |
|  |  | Performance | Low |
|  |  |  | Medium |
|  |  |  | High |
|  |  | Detection | Low |
|  |  |  | Medium |
|  |  |  | High |
|  |  | Attrition | Low |
|  |  |  | Medium |
|  |  |  | High |
|  |  | Reporting | Low |
|  |  |  | Medium |
|  |  |  | High |
|  | *Kranick et al., 2013* | Selection | Low |
|  |  |  | Medium |
|  |  |  | High |
|  |  | Performance | Low |
|  |  |  | Medium |
|  |  |  | High |
|  |  | Detection | Low |
|  |  |  | Medium |
|  |  |  | High |
|  |  | Attrition | Low |
|  |  |  | Medium |
|  |  |  | High |
|  |  | Reporting | Low |
|  |  |  | Medium |
|  |  |  | High |
|  | *Sperduti et al., 2014* | Selection | Low |
|  |  |  | Medium |
|  |  |  | High |
|  |  | Performance | Low |
|  |  |  | Medium |
|  |  |  | High |
|  |  | Detection | Low |
|  |  |  | Medium |
|  |  |  | High |
|  |  | Attrition | Low |
|  |  |  | Medium |
|  |  |  | High |
|  |  | Reporting | Low |
|  |  |  | Medium |
|  |  |  | High |
|  | *Wolpe et al., 2014* | Selection | Low |
|  |  |  | Medium |
|  |  |  | High |
|  |  | Performance | Low |
|  |  |  | Medium |
|  |  |  | High |
|  |  | Detection | Low |
|  |  |  | Medium |
|  |  |  | High |
|  |  | Attrition | Low |
|  |  |  | Medium |
|  |  |  | High |
|  |  | Reporting | Low |
|  |  |  | Medium |
|  |  |  | High |
|  | *Graham-Schmidt et al., 2016* | Selection | Low |
|  |  |  | Medium |
|  |  |  | High |
|  |  | Performance | Low |
|  |  |  | Medium |
|  |  |  | High |
|  |  | Detection | Low |
|  |  |  | Medium |
|  |  |  | High |
|  |  | Attrition | Low |
|  |  |  | Medium |
|  |  |  | High |
|  |  | Reporting | Low |
|  |  |  | Medium |
|  |  |  | High |
|  | *Saito et al., 2017* | Selection | Low |
|  |  |  | Medium |
|  |  |  | High |
|  |  | Performance | Low |
|  |  |  | Medium |
|  |  |  | High |
|  |  | Detection | Low |
|  |  |  | Medium |
|  |  |  | High |
|  |  | Attrition | Low |
|  |  |  | Medium |
|  |  |  | High |
|  |  | Reporting | Low |
|  |  |  | Medium |
|  |  |  | High |
|  | *Ricciardi et al., 2017* | Selection | Low |
|  |  |  | Medium |
|  |  |  | High |
|  |  | Performance | Low |
|  |  |  | Medium |
|  |  |  | High |
|  |  | Detection | Low |
|  |  |  | Medium |
|  |  |  | High |
|  |  | Attrition | Low |
|  |  |  | Medium |
|  |  |  | High |
|  |  | Reporting | Low |
|  |  |  | Medium |
|  |  |  | High |
|  | *Möller et al., 2020* | Selection | Low |
|  |  |  | Medium |
|  |  |  | High |
|  |  | Performance | Low |
|  |  |  | Medium |
|  |  |  | High |
|  |  | Detection | Low |
|  |  |  | Medium |
|  |  |  | High |
|  |  | Attrition | Low |
|  |  |  | Medium |
|  |  |  | High |
|  |  | Reporting | Low |
|  |  |  | Medium |
|  |  |  | High |
|  | *Zapparoli et al., 2020* | Selection | Low |
|  |  |  | Medium |
|  |  |  | High |
|  |  | Performance | Low |
|  |  |  | Medium |
|  |  |  | High |
|  |  | Detection | Low |
|  |  |  | Medium |
|  |  |  | High |
|  |  | Attrition | Low |
|  |  |  | Medium |
|  |  |  | High |
|  |  | Reporting | Low |
|  |  |  | Medium |
|  |  |  | High |
|  | *Finnemann et al., 2021* | Selection | Low |
|  |  |  | Medium |
|  |  |  | High |
|  |  | Performance | Low |
|  |  |  | Medium |
|  |  |  | High |
|  |  | Detection | Low |
|  |  |  | Medium |
|  |  |  | High |
|  |  | Attrition | Low |
|  |  |  | Medium |
|  |  |  | High |
|  |  | Reporting | Low |
|  |  |  | Medium |
|  |  |  | High |
|  | *Engel et al., 2022* | Selection | Low |
|  |  |  | Medium |
|  |  |  | High |
|  |  | Performance | Low |
|  |  |  | Medium |
|  |  |  | High |
|  |  | Detection | Low |
|  |  |  | Medium |
|  |  |  | High |
|  |  | Attrition | Low |
|  |  |  | Medium |
|  |  |  | High |
|  |  | Reporting | Low |
|  |  |  | Medium |
|  |  |  | High |

**PRISMA Checklist**

| **Section and Topic** | **Item #** | **Checklist item** | **Location where item is reported** |
| --- | --- | --- | --- |
| **TITLE** | | |  |
| Title | 1 | Identify the report as a systematic review. | 1 |
| **ABSTRACT** | | |  |
| Abstract | 2 | See the PRISMA 2020 for Abstracts checklist. | 2 |
| **INTRODUCTION** | | |  |
| Rationale | 3 | Describe the rationale for the review in the context of existing knowledge. | 3-5 |
| Objectives | 4 | Provide an explicit statement of the objective(s) or question(s) the review addresses. | 5 |
| **METHODS** | | |  |
| Eligibility criteria | 5 | Specify the inclusion and exclusion criteria for the review and how studies were grouped for the syntheses. | 5 |
| Information sources | 6 | Specify all databases, registers, websites, organisations, reference lists and other sources searched or consulted to identify studies. Specify the date when each source was last searched or consulted. | 5 |
| Search strategy | 7 | Present the full search strategies for all databases, registers and websites, including any filters and limits used. | 5 |
| Selection process | 8 | Specify the methods used to decide whether a study met the inclusion criteria of the review, including how many reviewers screened each record and each report retrieved, whether they worked independently, and if applicable, details of automation tools used in the process. | 5-6 |
| Data collection process | 9 | Specify the methods used to collect data from reports, including how many reviewers collected data from each report, whether they worked independently, any processes for obtaining or confirming data from study investigators, and if applicable, details of automation tools used in the process. | 5-6 |
| Data items | 10a | List and define all outcomes for which data were sought. Specify whether all results that were compatible with each outcome domain in each study were sought (e.g. for all measures, time points, analyses), and if not, the methods used to decide which results to collect. | 5-6 |
|  | 10b | List and define all other variables for which data were sought (e.g. participant and intervention characteristics, funding sources). Describe any assumptions made about any missing or unclear information. | 5-6 |
| Study risk of bias assessment | 11 | Specify the methods used to assess risk of bias in the included studies, including details of the tool(s) used, how many reviewers assessed each study and whether they worked independently, and if applicable, details of automation tools used in the process. | 6 |
| Effect measures | 12 | Specify for each outcome the effect measure(s) (e.g. risk ratio, mean difference) used in the synthesis or presentation of results. | 5-6 |
| Synthesis methods | 13a | Describe the processes used to decide which studies were eligible for each synthesis (e.g. tabulating the study intervention characteristics and comparing against the planned groups for each synthesis (item #5)). | 6 |
|  | 13b | Describe any methods required to prepare the data for presentation or synthesis, such as handling of missing summary statistics, or data conversions. | 6 |
|  | 13c | Describe any methods used to tabulate or visually display results of individual studies and syntheses. | 6 |
|  | 13d | Describe any methods used to synthesize results and provide a rationale for the choice(s). If meta-analysis was performed, describe the model(s), method(s) to identify the presence and extent of statistical heterogeneity, and software package(s) used. | 6 |
|  | 13e | Describe any methods used to explore possible causes of heterogeneity among study results (e.g. subgroup analysis, meta-regression). | 6 |
|  | 13f | Describe any sensitivity analyses conducted to assess robustness of the synthesized results. | 6 |
| Reporting bias assessment | 14 | Describe any methods used to assess risk of bias due to missing results in a synthesis (arising from reporting biases). | 6,8 |
| Certainty assessment | 15 | Describe any methods used to assess certainty (or confidence) in the body of evidence for an outcome. | N/A |
| **RESULTS** | | |  |
| Study selection | 16a | Describe the results of the search and selection process, from the number of records identified in the search to the number of studies included in the review, ideally using a flow diagram. | 7-15, 29, Suppl. |
|  | 16b | Cite studies that might appear to meet the inclusion criteria, but which were excluded, and explain why they were excluded. | 29, Suppl. |
| Study characteristics | 17 | Cite each included study and present its characteristics. | 7-20 |
| Risk of bias in studies | 18 | Present assessments of risk of bias for each included study. | Suppl. |
| Results of individual studies | 19 | For all outcomes, present, for each study: (a) summary statistics for each group (where appropriate) and (b) an effect estimate and its precision (e.g. confidence/credible interval), ideally using structured tables or plots. | N/A |
| Results of syntheses | 20a | For each synthesis, briefly summarise the characteristics and risk of bias among contributing studies. | 8 |
|  | 20b | Present results of all statistical syntheses conducted. If meta-analysis was done, present for each the summary estimate and its precision (e.g. confidence/credible interval) and measures of statistical heterogeneity. If comparing groups, describe the direction of the effect. | 19-20 |
|  | 20c | Present results of all investigations of possible causes of heterogeneity among study results. | 19-20 |
|  | 20d | Present results of all sensitivity analyses conducted to assess the robustness of the synthesized results. | N/A |
| Reporting biases | 21 | Present assessments of risk of bias due to missing results (arising from reporting biases) for each synthesis assessed. | N/A |
| Certainty of evidence | 22 | Present assessments of certainty (or confidence) in the body of evidence for each outcome assessed. | N/A |
| **DISCUSSION** | | |  |
| Discussion | 23a | Provide a general interpretation of the results in the context of other evidence. | 16-20 |
|  | 23b | Discuss any limitations of the evidence included in the review. | 19-20 |
|  | 23c | Discuss any limitations of the review processes used. | 19-20 |
|  | 23d | Discuss implications of the results for practice, policy, and future research. | 19-20 |
| **OTHER INFORMATION** | | |  |
| Registration and protocol | 24a | Provide registration information for the review, including register name and registration number, or state that the review was not registered. | N/A, 21 |
|  | 24b | Indicate where the review protocol can be accessed, or state that a protocol was not prepared. | N/A, 21 |
|  | 24c | Describe and explain any amendments to information provided at registration or in the protocol. | N/A, 21 |
| Support | 25 | Describe sources of financial or non-financial support for the review, and the role of the funders or sponsors in the review. | N/A, 20-21 |
| Competing interests | 26 | Declare any competing interests of review authors. | 20 |
| Availability of data, code and other materials | 27 | Report which of the following are publicly available and where they can be found: template data collection forms; data extracted from included studies; data used for all analyses; analytic code; any other materials used in the review. | N/A, Suppl. |
